# Scaling field pea technology in dryland areas through a cluster approach: The case of Waghimra zone, Ethiopia

**DOI:** 10.1371/journal.pone.0353275

**Published:** 2026-07-07

**Authors:** Ademe Mihiretu, Melaku Asresu, Kindye Ayen

**Affiliations:** 1 Amhara Regional Agricultural Research Institute, Sekota Dryland Agricultural Research Center, Sekota, Ethiopia; 2 Amhara Regional Agricultural Research Institute, Adet Agricultural Research Center, Bahir Dar, Ethiopia; King Saud University / Zagazig University, EGYPT

## Abstract

Adoption of improved crop technologies is widely recognized as essential for enhancing agricultural productivity, yet their validation under real farming conditions remains limited. The scaling trial was aimed to provide an interesting agronomic challenge through introducing a new technology that contradicts the established local field production practice. The trial was conducted over four consecutive production years (2019–2022) involving 109 (22 female) purposively selected farmers who managed a collective 32.5 ha of farmland. The farmers were strategically selected to promote the dissemination of new technologies, create demand, strengthen stakeholder linkages, and establish a sustainable technology multiplication system in the area. The comprehensive quantitative and qualitative data were collected and analyzed using proper statistical methods. The results demonstrated that the improved field pea technology was provided a 71.1% yield advantage compared to the existing local practices. With continuous expert support, 75% of farmers applied the full technology package, although 62.5% perceived it as labor-intensive, particularly during planting and thinning stages. Despite this, 95.9% of participants expressed a strong interest in cultivating the new technology in the future. Farmer-to-farmer diffusion was evident, with 1520 kg of improved seed distributed by 75% of involved farmers and stakeholders to non-participant but interested farmers. The farmers and stakeholders who attended different field days were also impressed and committed to adopting the new technology, recognizing its potential adaptation to moisture-deficient areas. These findings hence confirm the agronomic and social viability of the new technology. Therefore, it is recommended that scaling up and out of this improved field pea technology in similar areas be facilitated by the establishment of viable seed-multiplying cooperatives and strengthened stakeholder linkages.

## Introduction

Field pea *(Pisum sativum* L*.)* belongs to the family Fabaceae. It is one of the pulse crops grown in many countries and is considered the most important grain legume crop grown worldwide. Field pea serves as both human food and animal feed and is also regarded as a significant vegetable crop due to its high nutritional value and versatility [1,2]. It is grown in many countries and currently ranks fourth among the pulses in the world with a cultivated area of 6.33 million [3]. It is a cool-season pulse crop that adapts to a wide range of soil types from light sandy loam to heavy clay soils; however, it is sensitive to saline and waterlogged soil conditions which can adversely affect its growth and productivity [4,5].

In Ethiopia, field pea is an important crop for farmers’ livelihoods in the highlands; it provides food and feed, and a useful and cheap source of protein. It has a significant role in restoring soil fertility through rotation crops due to its notable nitrogen-fixation capacity, decreasing the need for fertilizer for low-income farmers [1,6–8]. It is widely grown from mid to high altitudes where annual rainfall ranges from 400 to 1000 mm. It ranks fourth in area coverage, reaching 220,194.82 ha with an annual production of 380,335.89 tons in Ethiopia [9–11]. On average 25147.7 ha of land has been allocated for field peas with a total average production of 21406 tons and an average yield of 1.73 tons ha^-1,^ which puts Ethiopia on the list of major field pea-producing countries in the world [9].

The wide distribution of the crop is attributed to its high protein content, well-balanced amino acid profile, high digestibility, and relatively higher yields compared to other pulse crops. It serves as an important source of protein for household consumption and provides income opportunities for resource-poor farmers [1]. Field pea grain is an inexpensive source of protein for the majority of Ethiopians with an annual estimated consumption of 6–7 kg per person [12]. In addition to providing dietary protein to the smallholder farmers, it is also cost-effective compared to animal protein, thus playing an important role in complementing cereal-based diets among resource-poor farmers [7]. The post-harvest by-products such as straw and pod walls of field peas are used for animal feed and farmers prefer the biomass for its good palatability quality for livestock.

The production and productivity of field pea in Ethiopia is constrained by biotic and abiotic factors. The major biotic factors include: susceptibility to pests and diseases, and weeding problems. Whereas the abiotic factors include a shortage of improved varieties, low-yielding potential land races, a lack of adaptable and early maturing varieties, soil fertility decline, and poor fertilizer utilization [8].

Field pea is the most important food legume next to faba bean in area coverage but following chickpea and mung bean in annual production in the Waghimra zone [9]. Although field peas are cultivated over extensive areas in the zone, overall production, productivity, and grain quality remain subpar due to the use of local and low-yielding varieties with suboptimal agronomic package utilization [6]. Farmers frequently cultivate field peas on infertile land with minimal or no tillage, employing broadcast sowing methods without implementing effective weed, pest and disease management strategies. All these limitations have hence caused lower productivity that is below regional, national, and international averages. Several agricultural research centres have released different varieties to enhance field pea production and productivity; however, the productivity of field peas is low due to insufficient evaluation and promotion of released and adapted varieties at the farmers’ level [13].

To alleviate the aforementioned production constraints, Sekota Dryland Agricultural Research Center had conducted a variety development trial to provide an adaptable, productive, and stable field pea variety for moisture stress areas of Waghimra. Improved field pea variety named ‘*Yewagnesh*’ with its full production package components was thus released in 2017, having superior performance across most agronomic traits [14]. To evaluate the variety under the farmers’ context, a comparative demonstration trial using the new field pea technology alongside the local production practice was made in 2018 using frontline farmers. The new field pea technology was hence ideal for scaling due to its high yield, economic feasibility, and preferred traits [15].

Based on the promising demonstration result, this scaling-up activity was conducted with the general objective of promoting the improved field pea technology to the wider community. Specifically, the study was intended to:

(i) introduce, transfer and diffuse the new field pea technology to smallholder farmers through wider demand creation,
(ii) create and strengthen linkage among the possible stakeholders in field pea production system, and
(iii) establishing a viable field pea technology multiplication and dissemination system in the study area and other similar agroeclogical zones.

## Materials and methods

### The study location

The Waghimra Zone is located in the northern part of Ethiopia consisting of eight districts in the Amhara region. It is characterized by its rugged topography, which has historically contributed to its susceptibility to natural challenges such as droughts. Soil erosion and truncated agricultural productivity are major problems in the study area. The major crops grown in these districts include sorghum, tef, wheat, barley, maize, faba bean, field pea, and chickpea [16]. The climate is predominantly classified as semi-arid, with significant spatial and temporal variability in rainfall. Among the five districts suitable for field pea cultivation, Dehana and Sekota were strategically selected for the current scaling trial due to their production potential and area coverage. In order to provide a collective climate variability picture, the annual rainfall and temperature patterns of the study districts were averaged across the four experimental seasons (Table 1). Farmers in these areas often cultivate field pea on infertile land with suboptimal agronomic practices such as minimal/no tillage, using local and low-yielding varieties, broadcast sowing, zero fertilizer input, and without any weed, pest, and disease management strategies.

**Table 1:** Geographic and climatic descriptions of experimental locations.

Districts	Location	Altitude	Rainfall	Temperate	Soil type
Dehana	12°55’59’‘N 38°42’93’‘E	2541 masl	895.2 mm	26.2°C	Black soil (vertisol)
Sekota	12°68’35’‘N 39.01’41’‘E	2100 masl	774.3 mm	28.5°C	Brown sandy soil

### Sampling and study design

The study was conducted over four experimental years in two districts, with one cluster established per district each year. In total, eight clusters were formed, covering 32.5 hectares of land. Farmer and farm selection followed a purposive sampling framework. Participants were included based on landholding within selected clusters, willingness to adopt the technology, and agreement to collaborate with district agricultural experts. The number of farmers per cluster varied according to landholding distribution, but all participants were purposively selected to ensure both agronomic feasibility and community readiness. The selection of farmlands and farmers was joint+ly undertaken with agricultural experts from district offices to ensure that the scaling initiative aligned with local agricultural priorities and technical standards.

### Farmer and cluster selection

Clusters were purposively selected based on crop rotation history to ensure compatibility with field pea cultivation, suitability for experimental management and production, as well as the farmers’ interest and willingness to participate in the trial and share the input costs. This purposive selection ensured that the chosen sites represented both agronomic feasibility and community readiness to adopt the technology. A total of 109 farmers (22 female) were included across the eight clusters in the four experimental years. Selection was conducted in collaboration with district agricultural experts, ensuring transparency and local relevance. The number of farmers per cluster was determined based on their landholding size within the cluster. Individual landholding sizes ranged from 0.25 to 1 hectare, with an average of 0.37 hectares per farmer. Landholding and crop management practices in both districts are predominantly managed by male-headed households, which limited female participation in the trial. However, the male-dominated participation was challenged by findings underscoring that gender disparities in landholding and decision-making limit women’s participation in agricultural innovation. Trials should adopt gender-transformative approaches rather than simply acknowledging the male dominance.

### Treatment design and procedures

The cluster-based scaling trial was conducted over four consecutive production years, from June 2019 to December 2022, across selected clusters to compare and assess changes among treatments. The study employed a cluster-based scaling trial approach to evaluate improved field pea technology under real farming conditions. This approach combined qualitative/participatory methods with a quantitative experimental design to address agronomic challenges in field pea production by introducing new technology that contrasts with existing traditional practices. In the study areas, farmers typically rely on traditional cultivars and agronomic practices, assuming minimal variance among varieties and production methods [17].

The field pea technology in this study involves the improved variety *‘Yewagnesh,’* accompanied by a comprehensive package of components, including recommended seed and fertilizer rates, optimal inter and intra-row spacing, land preparation, and weeding practices. The planting was executed in rows with seed and fertilizer rates of 150 kg ha^-1^ and 100 kg ha^-1^, respectively. Di-ammonium phosphate (DAP) fertilizer was applied through hand drilling, maintaining intra-row and inter-row spacing of 0.1m and 0.3m, respectively. Land preparation involved three rounds of plowing, and weeding was conducted twice, adhering to recommended practices [14].

In contrast, the local practice relies entirely on traditional methods, utilizing the existing field pea landrace named *‘Abeba ater’*, without fertilizer, broadcast sowing at a seed rate of 180 kg ha^-1^, and involves one/two plowing rounds with no weed management [15].

Before implementation, all participant farmers were provided training to create awareness on the new field pea technology practice, its package components and application as well as the entire research approach. Planting, tinning, weeding, follow-up, and joint monitoring as well as other management practices were done at the critical growth stage as required and recommended for all treatments.

### Data collection

Given that the participating farmers operated within the same cluster and shared a homogeneous socioeconomic background, variability was anticipated to be minimal. Consequently, in this study, 30% of the farmers were selected for interviews using a systematic sampling method with an interval of three from the comprehensive list of participant farmers (Eq. 1).


$$\begin{array}{c} K=\;\frac{N}{n} \end{array}$$ (1)

Where, K = sampling interval, N = scaling up participants, n = sample size

Prior to the beginning of the interview, participants were informed about the study, and verbal consent was obtained. The researcher then approached each selected participant and confirmed their willingness to voluntarily participate in the questionnaire process. This participatory perception-based scaling-up data collection was conducted among households with members aged 18 years and above; therefore, ethical approval was not required. The quantitative data on grain yield and straw biomass yield were collected, and the qualitative (social) data on farmers’ perceptions and attitudes toward the technology were collected.

In the study, both quantitative and qualitative data were collected using a semi-structured checklist, focus group discussions (FGDs), and a data sheet at the individual participant level. Quantitative data, such as grain and straw biomass yields, were collected at the individual participant farmer level using a semi-structured checklist. A comparable set of yield data was obtained from local practice plots located adjacent to the cluster, but they were managed alike by non-participant farmers, for comparative analysis. These plots adhered to the traditional field pea production practices, which included two rounds of tillage, broadcast sowing at a seed rate of 180 kg ha^-1^, and no application of fertilizers or weeding [15].

Qualitative primary data, including farmers’ preferences and perceptions of the technology, were gathered through face-to-face discussions and Likert scale items. Field days were organized across locations, involving farmers and other stakeholders, to evaluate the field pea technology under real settings.

### Data analysis

To account for seasonal variability, yield data from the cluster and adjacent farmlands were averaged over four years to provide a comprehensive overview. This methodology was essential as both new and local production practices were similarly influenced by environmental fluctuations. The collected yield data were analyzed using descriptive statistics, including mean, percentages, and frequency. To identify gaps in technology acceptance and application, the Extension Gap (EG) was calculated, indicating the yield barrier due to farmers’ lack of awareness in adopting improved technologies. The EG (Eq. 2) was determined by comparing yield data from scaling fields with yield records at the technology release and frontline demonstration stages [14,15].


$$\begin{array}{c} \text{EG}=\text{Dy}-\text{Fy} \end{array}$$ (2)

Where: Dy = demonstration yield at frontline farmers, Fy = farmers’ yield, EG = extension gap

However, relying on historical potential and demonstration data from secondary sources for EG estimation may introduce bias in identifying the sources of the gap and variation.

Farmers’ perceptions and demand for the new technology were evaluated using descriptive statistics by calculating the sum and average scores of various Likert scale items (Eq. 3, 4) [18]. An average score above 3.51 indicates a positive perception of the technology among farmers. Scores between 2.51 and 3.50 suggest a lack of confidence in the technology, while scores below 2.50 reflect a negative perception [19]. The internal consistency of the Likert-type questions was verified using Cronbach’s alpha. Constraints in the application of field pea technology were identified, described, and ranked [20]. Qualitative data from field days were presented through a thematic-oriented narration.


$$\begin{array}{c} \text{SS}=\sum_{i=\text{1}}^{\text{5}}\text{SD},\text{D},\text{ NAD},\text{ A},\text{ SA} \end{array}$$ (3)

Where, SS = sum of scores, SD = strongly disagree, D = disagree, NAD = neither agree nor disagree, A = agree, SA = strongly agree


$$\begin{array}{c} \text{Average score}=\;\frac{\text{Sum of score }}{\text{Sample size }} \end{array}$$ (4)

$$\begin{array}{c} \text{Percent position}=\;\frac{\text{1}\text{0}\text{0}\;(R_{ij}\;-\text{0}.\text{5})}{{\text{N}}_{\text{j}}} \end{array}$$ (5)

Where, R_ij_ = rank given for i^th^ constraint by j^th^ individual, N_j_ number of constraints ranked by j^th^ individual.

**Fig 1:**
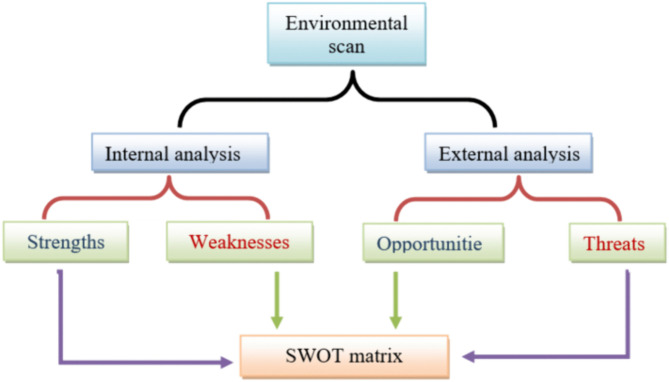
Theoretical framework of SWOT analysis adapted [19].

Moreover, a SWOT analysis was conducted utilizing an open-ended qualitative instrument to examine the external and internal environments of key stakeholders within the extension system [19]. This analysis aimed to identify stakeholders’ strengths and weaknesses, as well as opportunities and threats (Fig 1). Consequently, stakeholders can formulate strategies that leverage their strengths and mitigate weaknesses, while maximizing opportunities and neutralizing potential threats.

## Results and discussion

### Yield performance and technology gaps

A cluster-based scaling trial approach was employed to evaluate improved field pea technology under real farming conditions. The average grain yield (1540 kg ha^-1^) of the *‘Yewagnesh’* field pea had a 71.1% yield advantage over the conventional method of production (900 kg ha^-1^) in similar production years averaged across years. The straw yield (3610 kg ha^-1^) of improved technology also had a 30.3% advantage over the local practice (Table 2). The yield advantage of improved field pea technology over the traditional one might be due to the better adaptability and environmentally friendly nature of the new technology. This result is similar to the findings of Yadav et al. [21], who reported that location-specific mediation may have a massive impact on crop productivity improvement. The extension gap between the frontline demonstration plots and the farmers’ scaling-up land was recorded to be on average 361.7 kg ha^-1^. The extension gap in this study indicates that there is still room to increase field pea productivity through awareness creation and motivating farmers to adopt the recommended field pea production technology. Besides, the extension yield gaps are indicators of a lack of awareness of the adoption of improved farm technology by the farmers.

**Table 2:** Yield performance, productivity, and extension gap of field pea technology.

Field pea	Range yield index (kg ha^-1^)	Mean yield (kg ha^-1^)	SD	Yield index (%)	Extension gap (kg ha^-1^)
Grain	1180–1650	1540	14.787	71.1	361.7
Straw	2950–4180	3610	16.233	30.3	663.3

Note: Farmers’ grain (straw) yield of local field pea = 900 (2770) kg ha^-1^.

Grain (straw) yield of field pea during participatory evaluation trial = 1901.7 (4273.3) kg ha^-1.^

The higher average yield obtained from the scaling plots, compared with local practices, was primarily attributed to the adoption of the recommended variety and improved production practices. Conversely, the observed yield gap indicates the need to further encourage farmers to adopt the improved field pea production technologies instead of relying on existing local practices. This can be achieved through the use of diverse agricultural extension approaches and communication channels [22].

### Potential grain yield of Field pea = 2290 kg ha^-1^; SD = Standard Deviation. Farmers’ perception and demand for field pea technology

Most of the farmers had a positive view and good perception of the new field pea technology in most perception measurement parameters. The average score of 4.56 implies that the farmers perceived and accepted the technology with full confidence. The alpha coefficient (α = 0.76) demonstrated that the questions used to measure the farmers’ perception level were consistent and reliable. About 95.9% of participating farmers were highly interested in adopting the new field pea technology in the future, and most of them (72.5%) convinced their neighbor farmers to use the promising new technology (Table 3). Related findings also revealed that participatory varietal selection in South Asia improved farmer satisfaction and accelerated scaling of legumes, similar to the current cluster-based trial [23].

**Table 3:** Farmers’ perception of the new field pea technology.

Parameters	SD	D	NAD	A	SA	SS	MS
The germination performance of the crop is good.	--	--	–	47.2	52.8	245	4.50
The vegetative performance of the variety is good.	--	--	--	26.5	73.5	244	4.48
Seed setting performance of the variety is good.	--	--	16.1	27.1	56.8	248	4.53
The pod length of the variety is good.	--	–	3.9	18.7	77.4	244	4.48
The seed colour of the variety is good.	–	--	–	53.5	46.5	254	4.57
The variety is early maturing.	--	--	--	24.5	75.5	252	4.54
The variety is adaptable to marginal areas.	--	--	2.5	12.0	85.5	264	4.72
The productivity of the variety is good.	--	--	10	44.8	45.2	248	4.45
Seed per pod of the variety is good.	–	9.8	13.6	20.0	56.6	262	4.68
The food quality of the variety is good.	--	--	12.9	28.4	58.7	248	4.53
Interested in using new technology by next year.	--	--	4.1	35.4	60.5	278	4.52
Suggested that neighbors use the technology	–	--	27.5	20.2	52.3	284	4.78
			Average score = 4.56				
			Cronbach’s ‘α’ coefficient = 0.76				

Note: values are in percentage points (%); SD = strongly disagree, D = disagree, NAD = neither agree nor disagree, A = agree, SA = strongly agree, SS = Sum of scores, MS = Mean of scores.

Besides, field days were organized to disseminate the results, share responsibilities among stakeholders, and establish a sustainable system for scaling up improved technologies. Farmers, agricultural experts, NGOs, and journalists participated in the events. Stakeholders’ opinion regarding the visited field pea technology was summarized as follows:

*“The ‘Yewagnesh’ field pea technology is very adaptive to our dryland area and can give a yield under low moisture conditions than the existing varieties. Plus, it has an extra white colour which makes the variety marketable and demanded.”*

### Technology application and constraints

The applicability of new technology is a critical determinant of its adoption in smallholder farming systems. In this study, consistent expert support enabled about 75% of participating farmers to fully implement the improved field pea technology package. Nevertheless, adoption was not without challenges; thus, 62.5% of them perceived the technology as labor-intensive, mainly at planting and thinning stages. Thus, 71.9% of of them confronted labor shortages, often linked to small family sizes, female-headed households, as well as limited availability of eligible labor (Table 4). These findings highlight the importance of considering household demographics and labor dynamics when introducing new technologies.

**Table 4:** Farmers’ opinion on the applicability of field pea technology in the local context.

Variables	Indicators	Frequency	%
Did you apply the full technology package	Yes (No)	24 (08)	75 (25)
Do you think field pea production is labor-intensive?	Yes (No)	20 (12)	62.5 (37.5)
If yes, have you faced a labor shortage in applying?	Yes (No)	23 (09)	71.9 (28.1)
If yes, what are the possible reasons?	Smaller family size	13	40.7
	Female-headed	5	15.6
	Older household	5	15.6
Do you think row planting of field pea is difficult?	Yes (No)	16 (16)	50 (50)
If yes, what are the possible reasons?	Time-consuming	9	28.1
	Shortage of labor	4	12.5
	Lack of knowledge	3	9.4
Do you think the tinning of field peas is difficult?	Yes (No)	30 (02)	93.8 (6.2)
If yes, what are the possible reasons?	Lack of experience	12	37.5
	Cultural taboo	10	31.3
	Shortage of labor	8	25.0
Full technology application increased field pea production	Yes (No)	32 (00)	100 (00)

Specific practices, such as row sowing, were reported as difficult by 50% of respondents. Likewise, due to inexperience, cultural taboos, and labor shortages of participant farmers, nearly 93.8% of them experienced thinning difficulties (Table 4). Despite these constraints, the participant farmers collectively agreed that adherence to the recommended package significantly enhanced yield and yield components, underscoring the agronomic potential of the technology. These results resonate with findings that underline the role of participatory approaches in technology dissemination. Studies [23,24] demonstrate that the farmers’ willingness and interest are central to the farmers’ technology adoption and sustainable scaling, which supports the purposive selection framework used in this scaling trial. Similarly, other studies highlight that the resilience of farmer seed systems, which depend on farmer-driven diffusion and adaptation, is consistent with the peer-to-peer seed sharing observed in this study [25,26].

On the other hand, classic adoption studies [27,28] argue that technology scaling is constrained by structural factors such as access to credit, extension services, and risk perception. This suggests that selection based solely on landholding or chance may not adequately capture the key drivers of adoption. In addition, gender-focused research [22,17] underscores that male-dominated participation, as observed here, reflects broader inequalities in land access and decision-making power. Addressing these structural barriers through gender-transformative approaches is essential for ensuring equitable and sustainable adoption.

In general, while the improved field pea technology demonstrated strong agronomic performance and high farmer interest, adoption was moderated by labor constraints, cultural practices, and gender disparities. These findings highlight the need for scaling strategies that integrate participatory design, labor-saving innovations, and gender-transformative frameworks, thereby enhancing both the scientific value and practical applicability of new technologies in smallholder systems.

### Stakeholders’ linkage and technology exchange

Effective adoption of agricultural innovations depends not only on farmer participation but also on the strength of stakeholder linkages. Distributing responsibilities among farmers, extension services, and research institutions boosts interconnections and ensures sustainable promotion of new technologies [19]. In this study, agricultural experts at multiple levels provided continuous follow-up and consultation, which facilitated technology dissemination and improved collaboration compared to previous years. Identification of strengths, weaknesses, opportunities, and threats further highlights the importance of multi-actor engagement in scaling processes (Table 5).

**Table 5:** SWOT analysis summary of stakeholder linkage during scaling trial.

Strengths, Weaknesses, Opportunities, and Threats	Actors		
	E	R	F
Strengths:			
• Being an optimist and a higher demand for the new technology			Х
• Good contact throughout the scaling-up process	Х	Х	Х
• Sowing in clusters and according to the package			Х
• Availing inputs and training on time		Х	
• Collecting and analyzing the necessary data		Х	
Weaknesses:			
• Meager follow-up from nearby actors (local agricultural experts)	Х		
• Gap in full package application	Х		Х
• Problem in maintaining the seed quality			Х
• Stumpy technical backup to farmers		Х	
• Reluctant to weed and manage fields at the optimum level			Х
Opportunities:			
• The existence of NGOs working on agricultural technology promotion • Technology applications become the focus of the government • Farmers have good information about the improved technology • The existence of a seed exchange culture in the community (in any arrangement: cash, kind, free for non-eligible)			
Threats:			
• Dry land has the lowest and most erratic rainfall and high temperatures • High risk of drought within 5/7 years frequency • Low willingness to pay for input costs stemmed from the expense			

Note: E, F, and R stand for experts, farmers, and researchers, respectively.

These findings are consistent with international evidence emphasizing the role of participatory plant breeding and varietal selection in increasing adoption rates among smallholder farmers. Such approaches underscore that farmer willingness and interest are central to scaling, aligning with the purposive inclusion strategy applied in this cluster-based trial [23]. Moreover, the observed diffusion of improved seed among farmers reflects the dynamics of farmer seed systems, which rely on farmer-to-farmer exchange to sustain diversity and resilience.

At the same time, contrasting evidence from classic adoption studies [24] suggests that scaling is often constrained by structural factors such as access to credit, extension services, and risk perception. This indicates that purposive farmer selection based solely on landholding or willingness may not fully capture the broader drivers of adoption. Furthermore, gender-focused research [22] highlights persistent inequalities in land access and decision-making power, pointing to the need for gender-transformative frameworks that go beyond awareness to actively address structural barriers.

The strengthened stakeholder collaboration observed in this study provides a strong foundation for sustainable technology scaling. However, future interventions should incorporate participatory approaches, institutional support mechanisms, and gender-transformative strategies to ensure that technology adoption is equitable, inclusive, and resilient across diverse farming households.

**Fig 2:**
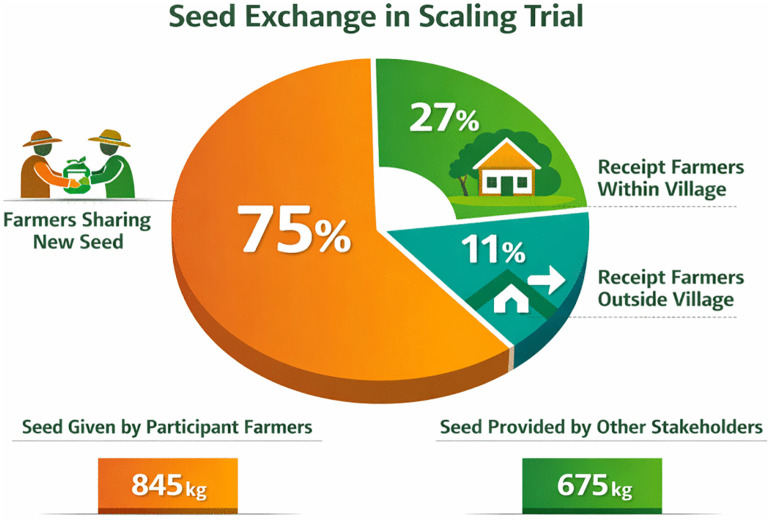
Seed exchange and the diffusion network of field pea technology.

Moreover, the solid seed exchange system takes the front line in the diffusion of improved varieties [19]. In this study, the amount of seed distributed by participant farmers and other stakeholders is illustrated below (Fig 2). The figure illustrated the diffusion of improved ‘Yewagnesh’ field pea seed among participant farmers and stakeholders. It showed that 75% of scaling-up farmers shared seed with others, including receipt farmers within (27%) and outside (11%) the village, highlighting strong farmer-to-farmer exchange. The graph also presented the amount of seed disseminated, i.e., 845 kg by participant farmers and 675 kg by other stakeholders, indicating the setting up of a robust seed exchange system that supports the sustainable technology scaling.

The current cluster-based trial closely aligns with international participatory approaches. The findings [23,22] indicated that farmer-led varietal selection accelerates scaling in smallholder systems. These findings support the participatory logic behind our trial, where the farmers’ willingness was central to technology dissemination. The study similarly highlights limitations in inclusive. Female participation was notably restricted, reflecting structural barriers in landholding and household labor dynamics. This challenge is consistent with global evidence on gender in agriculture [29], documenting persistent inequalities in land access and decision-making power, while [24] argued that agricultural interventions must adopt gender-transformative approaches to actively address these disparities.

As a result, while the seed exchange system and participatory design demonstrate strong potential for sustainable scaling, the trial also underscores the need for inclusive frameworks that integrate gender equity and structural support. Future scaling initiatives should combine farmer-led diffusion with institutional mechanisms such as seed cooperatives and gender-transformative extension strategies to ensure that adoption is both resilient and equitable across diverse farming households.

## Conclusion and recommendation

The cluster-based scaling trial demonstrated that the improved ‘*Yewagnesh’* field pea technology provided a substantial yield advantage over the traditional practices, confirming its agronomic potential under real farming circumstances. Strong farmer and stakeholder demand on the new technology was also generated through participatory engagement, though adoption was moderated by labor constraints, cultural practices, and gender disparities. These findings thus highlighted the importance of integrating participatory approaches, labor-saving innovations, and gender-transformative frameworks into scaling strategies to increase both scientific value and the practical technology applicability. Finally, we recommend that further dissemination of the new field pea technology to similar agro-ecologies be supported by targeted training and information provision. Sustainable adoption will also require strengthening seed-producing cooperatives, promoting stakeholder linkages, in addition to encouraging farmer-to-farmer diffusion through the existing extension networking (e.g., 1:5 systems at the grassroot village levels). Organizing closing workshops to connect stakeholders will be essential for devising pathways to scale the technology sustainably and ensure its wider community impact.

### Limitations

The current study basically aimed to provide farmers with information on the research output that visualizes the improved technology is by far better than the local production practice. Hence, for this purpose, data was collected from the undersigned experiment considering the existing practice as the control group which makes the comparison more complex beyond providing detailed information on the yield difference. In this case, performing inferential analysis may not sound as if the variation is by far visible. The authors hence suggest the need for more extensive data collection or additional research to understand the underlying treatment variations.
