# Supplementary material for: Scaling field pea technology in dryland areas through a cluster approach: The case of Waghimra zone, Ethiopia
Source: PLoS One. 2026 Jul 7;21(7):e0353275. doi: 10.1371/journal.pone.0353275 (PMC13340809; doi:10.1371/journal.pone.0353275)
Supplement: Attachment — Submitted filename: PONE-D-25-57361_reviewer-1_AB.pdf [file pone.0353275.s001.pdf]

PLOS ONE

Scaling Field Pea Technology in Dry-Land Areas through a Cluster Approach: The Case of Waghimra Zone, Ethiopia

--Manuscript Draft--

**Manuscript Number:**	PONE-D-25-57361
**Article Type:**	Research Article
**Full Title:**	Scaling Field Pea Technology in Dry-Land Areas through a Cluster Approach: The Case of Waghimra Zone, Ethiopia
**Short Title:**	Scaling Field Pea Technology in Dry-Land Areas
**Corresponding Author:**	Melaku Asresu<br>Amhara Regional Agricultural Research Institute<br>Bahir Dar, ETHIOPIA
**Keywords:**	Clustering approach, Extension gap, Farmer preference, Field Day, Yield advantage
**Abstract:**	Scaling up of improved field pea technology was carried out for four (2019-2022) consecutive production years in the potential woredas of the Wag-himra zone. A total of 109 farmers who had 32.5 ha of farmland in a cluster were selected to promote the new field pea technology by creating wider demand, strengthening linkage among the possible stakeholders as well and establishing a sustainable technology multiplication system. The required quantitative and qualitative data were collected and analyzed using proper statistical tools. The performance of improved technology has shown better performance and has a 71.1% yield advantage over the local field pea production practice. With the experts' continuous follow up about 75% of participant farmers applied the full technology package ; however, 62.5% of them believed that the new field pea technology was labor-intensive mainly in the planting and thinning stages. Out of the total participant farmers, 95.9% were highly interested in cultivating the new field pea technology in the future. Most farmers and stakeholders who attended the field days were fascinated and promised to use the new field pea technology as if it were adapted to their moisture deficit areas. About 75% of farmers and stakeholders diffused 1520 kg of improved field pea seed for interested fellow farmers. Therefore, we safely recommend the improved field pea technology for further scaling up/out to similar locations through establishing viable seed-multiplying cooperatives and stakeholder linkages.
**Order of Authors:**	Ademe Asresu Mihiretu
	Melaku Asresu
	Kindye Ayen
**Additional Information:**	
**Question**	**Response**
**Financial Disclosure**<br><br>Enter a financial disclosure statement that describes the sources of funding for the work included in this submission. Review the submission guidelines for detailed requirements. View published research articles from *PLOS ONE* for specific examples.<br><br>This statement is required for submission and **will appear in the published article** if the submission is accepted. Please make	The author(s) received no specific funding for this work.

Powered by Editorial Manager® and ProduXion Manager® from Aries Systems Corporation

sure it is accurate.<br><br>**Funded studies**<br>Enter a statement with the following details:<br>• Initials of the authors who received each award<br>• Grant numbers awarded to each author<br>• The full name of each funder<br>• URL of each funder website<br>• Did the sponsors or funders play any role in the study design, data collection and analysis, decision to publish, or preparation of the manuscript?<br><br>Did you receive funding for this work?	
**Competing Interests**<br><br>Use the instructions below to enter a competing interest statement for this submission. On behalf of all authors, disclose any competing interests that could be perceived to bias this work—acknowledging all financial support and any other relevant financial or non-financial competing interests.<br><br>This statement is required for submission and **will appear in the published article** if the submission is accepted. Please make sure it is accurate and that any funding sources listed in your Funding Information later in the submission form are also declared in your Financial Disclosure statement.<br><br>View published research articles from *PLOS ONE* for specific examples.	The authors have declared that no competing interests exist.

**NO authors have competing interests**<br><br>Enter: *The authors have declared that no competing interests exist.*<br><br>**Authors with competing interests**<br><br>Enter competing interest details beginning with this statement:<br><br>*I have read the journal's policy and the authors of this manuscript have the following competing interests: [insert competing interests here]*<br><br>* typeset	
**Ethics Statement**<br><br>Enter an ethics statement for this submission. This statement is required if the study involved:<br><br>• Human participants<br>• Human specimens or tissue<br>• Vertebrate animals or cephalopods<br>• Vertebrate embryos or tissues<br>• Field research<br><br>Write "N/A" if the submission does not require an ethics statement.<br><br>General guidance is provided below. Consult the submission guidelines for detailed instructions. **Make sure that all information entered here is included in the Methods section of the manuscript.**	Verbal informed consent was obtained from participants that the information will be used anonymously for publication.

Powered by Editorial Manager® and ProduXion Manager® from Aries Systems Corporation

**Format for specific study types**<br><br>**Human Subject Research (involving human participants and/or tissue)**<br>• Give the name of the institutional review board or ethics committee that approved the study<br>• Include the approval number and/or a statement indicating approval of this research<br>• Indicate the form of consent obtained (written/oral) or the reason that consent was not obtained (e.g. the data were analyzed anonymously)<br><br>**Animal Research (involving vertebrate animals, embryos or tissues)**<br>• Provide the name of the Institutional Animal Care and Use Committee (IACUC) or other relevant ethics board that reviewed the study protocol, and indicate whether they approved this research or granted a formal waiver of ethical approval<br>• Include an approval number if one was obtained<br>• If the study involved *non-human primates*, add *additional details* about animal welfare and steps taken to ameliorate suffering<br>• If anesthesia, euthanasia, or any kind of animal sacrifice is part of the study, include briefly which substances and/or methods were applied<br><br>**Field Research**<br><br>Include the following details if this study involves the collection of plant, animal, or other materials from a natural setting:<br>• Field permit number<br>• Name of the institution or relevant body that granted permission	
**Data Availability**<br><br>Authors are required to make all data underlying the findings described fully available, without restriction, and from the time of publication. PLOS allows rare exceptions to address legal and ethical concerns. See the PLOS Data Policy and FAQ for detailed information.	Yes - all data are fully available without restriction

A Data Availability Statement describing where the data can be found is required at submission. Your answers to this question constitute the Data Availability Statement and **will be published in the article**, if accepted.<br><br>**Important:** Stating 'data available on request from the author' is not sufficient. If your data are only available upon request, select 'No' for the first question and explain your exceptional situation in the text box.<br><br>Do the authors confirm that all data underlying the findings described in their manuscript are fully available without restriction?	
**Describe where the data may be found in full sentences. If you are copying our sample text, replace any instances of XXX with the appropriate details.**<br><br>• If the data are **held or will be held in a public repository**, include URLs, accession numbers or DOIs. If this information will only be available after acceptance, indicate this by ticking the box below. For example: *All XXX files are available from the XXX database (accession number(s) XXX, XXX.).*<br>• If the data are all contained **within the manuscript and/or Supporting Information files**, enter the following: *All relevant data are within the manuscript and its Supporting Information files.*<br>• If neither of these applies but you are able to provide **details of access elsewhere**, with or without limitations, please do so. For example:<br><br>*Data cannot be shared publicly because of [XXX]. Data are available from the XXX Institutional Data Access / Ethics Committee (contact via XXX) for researchers who meet the criteria for access to confidential data.*<br><br>*The data underlying the results presented in the study are available from (include the name of the third party*	All relevant data are within the paper

and contact information or URL).<br>• This text is appropriate if the data are owned by a third party and authors do not have permission to share the data.<br><br>* typeset	
Additional data availability information:	

Manuscript Click here to access/download;Manuscript;Plose Field pea main.docx

Scaling Field Pea Technology in Dry-Land Areas through a Cluster Approach: The Case of Waghimra Zone, Ethiopia

Ademe Mihiretu[1], Melaku Asresu[1] and Kindye Ayen[2]

[1] Socio-economics and Agricultural Extension, Sekota Dryland Agricultural Research Center, Sekota, Ethiopia

[2] Socio-economics and Agricultural Extension, Adet Agricultural Research Center, Amhara regional Agricultural Research Institute, Bahir Dar, Ethiopia

*Corresponding Author: masresu5@gmail.com

Abstract

Scaling up of improved field pea technology was carried out for four (2019-2022) consecutive production years in the potential woredas of the Wag-himra zone. A total of 109 farmers who had 32.5 ha of farmland in a cluster were selected to promote the new field pea technology by creating wider demand, strengthening linkage among the possible stakeholders as well and establishing a sustainable technology multiplication system. The required quantitative and qualitative data were collected and analyzed using proper statistical tools. The performance of improved technology has shown better performance and has a 71.1% yield advantage over the local field pea production practice. With the experts' continuous follow up about 75% of participant farmers applied the full technology package; however, 62.5% of them believed that the new field pea technology was labor-intensive mainly in the planting and thinning stages. Out of the total participant farmers, 95.9% were highly interested in cultivating the new field pea technology in the future. Most farmers and stakeholders who attended the field days were fascinated and promised to use the new field pea technology as if it were adapted to their moisture deficit areas. About 75% of farmers and stakeholders diffused 1520 kg of improved field pea seed for interested fellow farmers. Therefore, we safely recommend the improved field pea technology for further scaling up/out to similar locations through establishing viable seed-multiplying cooperatives and stakeholder linkages.

Keywords: Clustering approach, Extension gap, Farmer preference, Field day, Yield advantage

Introduction

Field pea *(Pisum sativum* L.*)* is among the pulse crops grown in many countries of the world. In Ethiopia, it is widely grown from mid to high-altitude ranges and ranks fourth in area coverage and annual production (Kandel et al., 2016). Field pea is grown in many countries and currently ranks fourth among the pulses in the world with a cultivated area of 6.33 million (Tamene et al., 2013). It is widely grown from mid to high altitudes and ranks fourth in area coverage, reaching 212,890 ha with an annual production of 2,632,663.9 tons in Ethiopia (Mulusew et al., 2010). On average 25147.7 ha of land has been allocated for field peas with a total average production of 21406 tons and an average productivity of 0.86 ton ha^{-1} which puts Ethiopia on the list of major field pea producing countries in the world (CSA, 2016).

Grain is an inexpensive source of protein for the majority of Ethiopians with an annual estimated consumption of 6-7 kg per person (MOARD, 2015). The post-harvest by-products such as straw and pod walls of field peas are used for animal feed. Field pea is the most important food legume next to faba bean in area coverage, but following faba bean and chickpea in annual production in the Wag-himra zone (Cherinet and Tazebachew, 2015).

Therefore, there was a need to supply a variety that is adaptable, productive and suitable to moisture stress areas by identifying the best performing and stable genotype in terms of yield and other traits for Wag-lasta areas. An improved field pea variety named '*Yewagnesh*' was thus released for the region in 2017 achieving the most agronomic traits (Yirga *et al.*, 2019). Demonstration of improved field pea variety alongside the local cultivar with and without the full package was made under the participation of farmers and experts. The improved field pea variety under a full production package (Field pea technology[1], hereafter) was ideal for further promotion due to its productivity, economic feasibility as well as farmers' and experts' selection (Mihiretu and Assefa, 2019).

Based on the promising demonstration result, this scaling-up activity was planned with the general objective of promoting the improved field pea technology to the wider community. More specifically, the study was intended to transfer the recommended field pea technology to the interested smallholder farmers by creating wider demand, strengthening linkage among the

[1]Improved variety with its full production package which includes a comprehensive set of practices such as recommended fertilizer rates, inter and intra-row spacing, land preparation, and weed management.

possible stakeholders as well as establishing a viable technology multiplication and dissemination system.

Materials and Methods

Farmers selection, training and technology implementation

The scaling-up activity was conducted over four consecutive production years from June 2019 to December 2022 in the potential districts of Dahana and Sekota in the Wag-himra Zone. A total of 109 (22 female) farmers who had 32.5ha of farmland in a cluster were selected to participate in the scaling-up. The improved *'Yewagnesh'* field pea variety with its full production package was promoted. Researchers and Development Agents together have selected the participants and measured their clustered field. Before implementation, all participant farmers were provided training on basic agronomic practices to create awareness about the field pea technology and the extension approach as a whole. Planting was done in a row at a 150 kg ha^{-1} seed rate as per the recommendation. NPS fertilizer was applied at the rate of 100 kg ha^{-1}, but weeding and other management practices were done as required (Yirga et al., 2019).

The major stakeholders working on agricultural development at different levels were identified in the respective areas to be involved and made continuous follow-up in the scaling-up process. Continious follow-up and joint monitoring and evaluation were made following the critical plant physiological stages. Field days were organized in all locations involving farmers and other stakeholders to evaluate the field pea technology under the farmers' real conditions and promote it for the wider community (Russell, 1997).

Data collection and analysis

In order to collect the farmers' opinions and perceptions about the new technology a semi-structured checklist was used. In the study out of the total participant farmers, about 30% were selected for the interview using systematic sampling by an approximate sampling interval of 3 (Eq. 1).

$$K = \frac{N}{n} \qquad (1)$$

Where, K = sampling interval, N = pre-scaling up participants, n = sample size

Data on grain and straw yields of the scaling-up field and the nearby adjacent farms were collected. The yield and related agronomic data of the prior demonstration study were taken from published articles by Mihiretu and Assefa (2019) to calculate the extension gap (EG).

$$EG = Dy - Fy \quad \ldots\ldots \quad (2)$$

Where: Dy=demonstration yield at frontline farmers, Fy=farmers' yield, EG=extension gap

Finally, the farmers' perception and demand for the new technology were assessed in descriptive statistics by calculating the sum and average scores[2] (Eq. 3, 4) of different Likert scale items (Ferdous et al, 2017). The Cronbach's alpha was checked for internal consistency among Likert-type questions. Constraints in field pea technology application were described and ranked (Russell, 1997). The positive and negative sides of stakeholders involved in field pea pre-scaling up were assessed via SWOT (strengths, weaknesses, opportunities, threats) analysis (Mihiretu, 2019). The qualitative field day data were explained in a thematic-oriented narration.

$$SS = \sum_{i=1}^{5} SD, D, NAD, A, SA \quad \ldots\ldots \quad (3)$$

Where, SS = sum of scores, SD = strongly disagree, D = disagree, NAD = neither agree nor disagree, A = agree, SA = strongly agree

$$\text{Average score} = \frac{\text{Sum of score}}{\text{Sample size}} \quad \ldots\ldots \quad (4)$$

$$\text{Percent position} = \frac{100\ (R_{ij} - 0.5)}{N_j} \quad \ldots\ldots \quad (5)$$

Where, R_{ij} = rank given for i^{th} constraint by j^{th} individual, N_j number of constraints ranked by j^{th} individual.

Results and Discussion

Yield performance and gaps of field pea technology

The average grain yield (1540 kg ha^{-1}) of *the 'Yewagnesh'* field pea had a 71.1% yield advantage over the conventional method of production (900 kg ha^{-1}) in similar production years. The straw yield (3610 kg ha^{-1}) of improved technology also had a 30.3% advantage over the local practice (Table 1). The yield increase of improved field pea technology over the traditional one might be due to the better adaptability and environmentally friendly nature of the new technology. This

[2] If the average score is greater than 3.51, the farmers have a good perception of the technology. If the average score is 2.51-3.50, the farmers have no confidence in the technology. If the average score is below 2.50, the farmers do have not a good perception of the technology (Mihiretu, 2019).

result is similar to the findings of Yadav et al. (2021), who reported that location-specific mediations may have a massive impact on crop productivity improvement. The extension gap between the frontline demonstration plots and the farmers' scaling-up land was recorded to be on average 361.7 kg ha^{-1}. The extension gap in this study indicates that there is still room to increase field pea productivity through awareness creation and motivating farmers to adopt the recommended field pea production technology. Besides, the extension yield gaps are indicators of a lack of awareness of the adoption of improved farm technology by the farmers.

Table 1. Yield performance, productivity and extension gap of field pea technology

Field pea	Range yield index (kg ha^{-1})	Mean yield (kg ha^{-1})	SD	Yield index (%)	Extension gap (kg ha^{-1})
Grain	1180 – 1650	1540	14.787	71.1	361.7
Straw	2950 – 4180	3610	16.233	30.3	663.3

Note: Farmers' grain (straw) yield of local field pea = 900 (2770) kg ha^{-1}
Grain (straw) yield of Field pea during participatory evaluation trial = 1901.7 (4273.3) kg ha^{-1}
Potential grain yield of Field pea = 2290 kg ha^{-1}; SD = Standard Deviation

Farmers' perception and demand for field pea technology

Most of the farmers had a positive view and good perception of the new field pea technology in most perception measurement parameters. The average score of 4.56 implies that the farmers perceived and accepted the technology with full confidence. The alpha coefficient (α=0.76) demonstrated that the questions used to measure the farmers' perception level were consistent and reliable. About 95.9% of participating farmers were highly interested in adopting the new field pea technology in the future, and most of them (72.5%) convinced their neighbor farmers to use the promising new technology (Table 2).

Besides, field days involving farmers, agricultural experts, NGOs and journalists were held and stakeholders' opinion about the visited field pea technology was summarized as follows:

> *"The 'Yewagnesh' field pea technology is very adaptive to our dry-land area and can give yield under low moisture than the existing varieties. Plus, it has an extra white colour which makes the variety marketable and demanded."*

Table 2. Farmers' perception of the new field pea technology

Parameters	SD	D	NAD	A	SA	SS	MS
The germination performance of the crop is good.	--	--	-	47.2	52.8	245	4.50
The vegetative performance of the variety is good.	--	--	--	26.5	73.5	244	4.48
Seed setting performance of the variety is good.	--	--	16.1	27.1	56.8	248	4.53

The pod length of the variety is good.	--	-	3.9	18.7	77.4	244	4.48
The seed colour of the variety is good.	-	--	-	53.5	46.5	254	4.57
The variety is early maturing.	--	--	--	24.5	75.5	252	4.54
The variety is adaptable to marginal areas.	--	--	2.5	12.0	85.5	264	4.72
The productivity of the variety is good.	--	--	10	44.8	45.2	248	4.45
Seed per pod of the variety is good.	-	9.8	13.6	20.0	56.6	262	4.68
The food quality of the variety is good.	--	--	12.9	28.4	58.7	248	4.53
Interested in using new technology by next year.	--	--	4.1	35.4	60.5	278	4.52
Suggested for neighbors to use the technology	-	--	27.5	20.2	52.3	284	4.78
							Average score = 4.56
							Cronbach's 'α' coefficient = 0.76

Note: values are in percentage points (%); SD = strongly disagree, D = disagree, NAD = neither agree nor disagree, A = agree, SA= strongly agree, SS = Sum of scores, MS = Mean of scores

Technology application and constraints

Among other factors, applicability plays a significant role in new technology adoption; therefore, with the experts' continuous follow up about 75% of participant farmers applied the full technology package of the improved field pea. Of the total respondents, however, 62.5% believed that the new field pea production technology was labor-intensive, especially in the planting and thinning stages. About 71.9% of them faced labor shortages due to factors like a small family size, being female-headed and older households in descending order resulting in a lack of eligible family members (Table 3). Similarly, half of the respondents said that row planting of field peas was difficult if it is time-consuming, on top of a shortage of labor and lack of knowledge in descending order. On the other hand, 93.8% of the total respondents mentioned that tinning the field pea plant is so difficult because of a lack of experience, cultural taboos and shortage of labor. Nonetheless, respondents as a whole agreed that producing field peas as per the technology recommendation is very helpful in increasing yield and yield components.

Table 3. Farmers' opinion on the applicability of field pea technology in the local context

Variables	Indicators	Frequency	%
Did you apply the full technology package	Yes (No)	24 (08)	75 (25)
Do you think field pea production is labor-intensive?	Yes (No)	20 (12)	62.5 (37.5)
If yes, have you faced a labor shortage in applying?	Yes (No)	23 (09)	71.9 (28.1)
If yes, what are the possible reasons?	Smaller family size	13	40.7
	Female-headed	5	15.6
	Older household	5	15.6
Do you think row planting of field pea is difficult?	Yes (No)	16 (16)	50 (50)
If yes, what are the possible reasons?	Time-consuming	9	28.1
	Shortage of labor	4	12.5
	Lack of knowledge	3	9.4
Do you think the tinning of field peas is difficult?	Yes (No)	30 (02)	93.8 (6.2)
If yes, what are the possible reasons?	Lack of experience	12	37.5
	Cultural taboo	10	31.3
	Shortage of labor	8	25.0
Full technology application increased field pea production	Yes (No)	32 (00)	100 (00)

Stakeholders' linkage and technology exchange

Sharing duties among stakeholders would consolidate the triple linkages of farmers-extension-research, and that is in turn vital to sustainable technology promotion and adoption (Mihiretu, 2019). The agricultural experts at different levels were also handling tasks to facilitate the technology dissemination via continuous follow-up and consultation. The cooperation among concerned actors in the promotion process increased compared to the preceding years. Therefore, the strengths, weaknesses, opportunities, and threats of all actors involved in the scaling-up process were listed and defined below in Table 4.

Table 4. SWOT analysis of stakeholder linkage

Strengths, Weaknesses, Opportunities and Threats	Actors		
	E	R	F
**Strengths:**			
▪ Being an optimist and a higher demand for the new technology			X
▪ Good contact throughout the pre-scaling-up process	X	X	X
▪ Sowing in cluster and according to the package			X
▪ Availing inputs and training on time		X	
▪ Collecting and analyzing the necessary data		X	
**Weaknesses:**			
• Meager follow-up from nearby actors (local agricultural experts)	X		
• Gap in full package application	X		X
• Problem in maintaining the seed quality			X
• Stumpy technical backup to farmers		X	
• Reluctant to weed and managing fields at the optimum level			X
**Opportunities:**			
▪ The existence of NGOs working on agricultural technology promotion			
▪ Technology applications become the focus of the government			
▪ Farmers have good information about the improved technology			
▪ The existence of seed exchange culture in the community (in any arrangement: cash, kind, free for non-eligible)			
**Threats:**			
▪ Dry land has the lowest and erratic rainfall and high temperature			
▪ High risk of drought within 5/7 years frequency			
▪ Low willingness to pay for input costs stemmed from expensiveness			

Note: E, F and R stands for experts, farmers, and researchers, respectively.

Moreover, the solid seed exchange system takes the front line in the diffusion of improved varieties (Mihiretu, 2019). In this study, 75% of scaling-up participant farmers were handed over the new field pea technology to interested fellow farmers in different arrangements. The amount of seed disseminated to interested farmers in/outside the study areas by participant farmers and other stakeholders is presented below (Table 5).

Table 5. Seed exchange and the diffusion network of field pea technology

	Farmers who shared the new seed	Receipt farmers within the village	Receipt farmers outside the village	Amount of seed exchanged by participant farmers in kg.	Seed exchanged by other stakeholders in kg
Freq.	24	38	16	845	675
%	75				

Conclusion and recommendation

The scaling-up result confirmed that the improved field pea technology provided a better yield and advantage than the existing production practice both in grain and straw yields. Additionally, strong demand for the new field pea technology was created by farmers and other stakeholders during field days. Skill gap, the complexity of the new technology and being pessimist about the new technology were the top three challenges experienced by farmers to apply the full technology package in field pea production. The authors, therefore, recommend that the improved '*Yewagnesh'* field pea variety with its full production package should further scale out to similar agro-ecologies by providing appropriate training and information about the technology. Finally, organizing closing workshops linking stakeholders is worthwhile to devise ways to forward the technology to the wider community in a sustainable manner. In this regard, farmers are advised to use the usual 1:5 extension networking to hand over the technology to interested fellow farmers very easily. Seed-producing and marketing cooperatives should strengthen to make field pea technology multiplication and transfer sustainable.

Acknowledgment

The authors would like to acknowledge the farmers and local experts who made magnificent efforts in comprehending the research.

Data Availability: The data of this study will be available on reasonable request to the corresponding author

Funding: The authors received no specific funding for this study.

Conflict of interest: The authors have no conflict of interest

Consent for publication

Verbal informed consent was obtained from participants that the information will be used anonymously for publication.

References

Central Statistical Agency (CSA). (2016). Agricultural Sample Survey. 2015/2016 Volume I Report on Area and Production of Major Crops. Statistical Bulletin, May 2015/16. Addis Ababa.

Cherinet, A. and Tazebachew, A. (2015). Adaptability of Field pea (*Pisum sativum L.)* varieties under Irrigation at the Western Amhara Region, Ethiopia. International Journal of Plant Breeding and Genetics, 9, 28-31.

Ferdous Z, Datta A, Anwar M (2017). Effects of plastic mulch and indigenous microorganism on yield and yield attributes of cauliflower and tomato in inland and coastal regions of Bangladesh. J. Crop Improv. 31:261-279.

Kandel, H., McPhee, K., Akyüz, A., Main, N., Schatz, B., Zwinger, S., … Jacobs, J. (2016). North Dakota Dry Pea Variety Trial Results for 2016 and Selection Guide. NDSU Extension Service.

Mihiretu, A. (2019). Participatory evaluation and promotion of improved Bread wheat technology in the dry lands of Wag-lasta, Ethiopia: challenges and Prospects. *J. Ext. Educ,* 31 (4), 6370–6380. https://doi.org/10.26725/JEE.2019.4.31.6370-6380

Mihiretu, A. and N. Assefa, “Comparative evaluation and demonstration of field pea production practices in intermediate altitudes of Northeastern Amhara, Ethiopia,” Turkish Journal of Agriculture - Food Science and Technology, vol. 7, no. 11, pp. 1901–1907, 2019.

MOARD (Ministry of Agriculture and Rural Development). 2015. Crop Variety Register, 3(11), 68-73, Addis Ababa, Ethiopia.

Mulusew, F., Tadele, T., Setegn, G. and Bekele, H. (2010). Agronomic performances, disease reaction and yield stability of field pea *(Pisum sativum L.)* genotypes in Bale Highlands, Ethiopia. Australian Journal of Crop Science, 4(4), 238–246.

Russell. (1997). Pairwise ranking made easy. In: PLA notes No 28, Methodological Complementary. International Institute of Environmental and Development (IIED), London, pp. 25-27.

Tamene, T., Yeneneh, B., Tadess, T., Mussa, J. and Gemechu, K. (2013). Genotype×Environment Interaction and Performance Stability for Grain Yield in Field Pea *(Pisum sativum L.)* Genotypes. International Journal of Plant Breeding.

Yadav DB, Kamboj BK,Garg RB (2021). Increasing the productivity and profitability of sunflowers through front-line demonstrations in the irrigated agro-ecosystem of eastern Haryana. Haryana J. Agron. 20(1-2):3335.

Yirga, KI, Aemiro BE, Wubeshet BE., Zinabu NI, Zelalem AS, Antenh AD, Birke TE, Genet KE, Tesfay AL, Fentaw AS. 2019. Field Pea (Pisum sativum L.) Variety Development for Moisture Deficit Areas of Eastern Amhara, Ethiopia. Hindawi Advances in Agriculture, 1398612 (6).
