# Supplementary material for: Scaling field pea technology in dryland areas through a cluster approach: The case of Waghimra zone, Ethiopia
Source: PLoS One. 2026 Jul 7;21(7):e0353275. doi: 10.1371/journal.pone.0353275 (PMC13340809; doi:10.1371/journal.pone.0353275)
Supplement: Attachment — Submitted filename: Response to Reviewers.docx [file pone.0353275.s003.docx]

**Point by point response to reviewers’ questions and comments**

**Academic editor:**

Please respond carefully for all reviewers comments?

**Responses to Academic editor**

We have carefully addressed the reviewers’ comments by strengthening the comparative discussion with worldwide studies, clarifying methodological details, and refining the presentation of results, limitations, and conclusions.

**Reviewer #1:**

The manuscript has undergone a thorough revision compared to the first, original manuscript. I received satisfactory answers to my formulated questions. Thus, the value of the article has increased significantly. Although I still maintain that the scientific value and uniqueness of the manuscript are less prominent and its literary support and supplementation are needed, and further confrontations with existing international studies would also increase the value of the work.

**Responses to Reviewer # 1**

We appreciate the reviewer’s observation that the scientific value and uniqueness of the manuscript could be strengthened. We recognize the importance of situating our findings within the broader body of international literature to enhance the contribution of this work.

In response, we have revised the manuscript to include additional references to relevant international studies on field pea technology scaling and participatory agricultural trials though limited in number. This supplementation provides a stronger comparative framework and highlights how our findings align with, diverge from, or extend existing knowledge.

We have also emphasized the unique aspects of our study, particularly the cluster-based participatory design implemented under real farming conditions in Ethiopia. This approach contributes novel insights into farmer-driven scaling initiatives in smallholder contexts, which are less represented in international literature.

The revised discussion section now explicitly confronts existing international studies, drawing out methodological similarities and differences. This comparative analysis underscores the scientific value of our findings by situating them within global debates on technology adoption, gender participation, and smallholder scaling trials. Therefore, we have supplemented the manuscript with additional citations and theoretical framing to strengthen the academic rigor and ensure that the work is well-supported by existing scholarship.

**Reviewer #3:**

My concerns are still on the description of farmer and farm selection, which remains unclear and methodologically inconsistent. The manuscript alternates between purposive selection, “determination by chance,” and criteria such as accessibility, farmer interest, and budget constraints, without clearly defining the sampling framework. This creates ambiguity regarding whether the study follows a qualitative/participatory approach or a quantitative experimental design. I am really struggling to place it from reading sections under lines 107 and 121. As currently presented, the methodological basis for farmer selection and study design is insufficiently justified. While the work has merit and could contribute meaningfully to the literature on technology scaling under real farming conditions, significant revision is required to address these methodological and presentation concerns.

**Responses to Reviewer # 3**

We acknowledge the reviewer’s concern regarding the apparent inconsistency in describing farmer and farm selection.

However, in the revised manuscript, we have clarified that the study employed a purposive sampling approach. Clusters and farmers were selected based on predefined criteria (crop rotation history, accessibility, suitability, and farmer interest). The phrase “determined by chance” has been removed to avoid confusion, and the methodology is now consistently described as purposive and participatory.

**Justification on the methodological choice:** Purposive sampling was chosen over random sampling because the trial aimed to evaluate technology adoption under realistic farming conditions. Farmer willingness to participate and ability to share input costs were essential for implementation. Thus, the design reflects a participatory scaling trial rather than a purely randomized experimental design.

**Explanation on the farmers’ distribution:** The variation in the number of farmers per cluster was not due to random allocation but rather to differences in landholding sizes within clusters. We have revised the text to state: *“The number of farmers per cluster varied according to landholding distribution, but all participants were purposively selected based on eligibility criteria.”* This ensures methodological consistency.

Therefore, we have clarified that the study follows a participatory, cluster-based scaling trial design. While quantitative data were collected, the primary emphasis was on evaluating technology adoption in real-world settings, which necessitated purposive inclusion of farmers rather than random assignment.
