# Supplementary material for: Scaling field pea technology in dryland areas through a cluster approach: The case of Waghimra zone, Ethiopia
Source: PLoS One. 2026 Jul 7;21(7):e0353275. doi: 10.1371/journal.pone.0353275 (PMC13340809; doi:10.1371/journal.pone.0353275)
Supplement: Attachment — Submitted filename: Response to Reviewers 2026.docx [file pone.0353275.s002.docx]

**Date**: 15/03/2026

**Author Response to Reviewers**
**Manuscript Title**: Scaling Field Pea Technology in Dry-Land Areas through a Cluster Approach: The Case of Waghimra Zone, Ethiopia (PONE-D-25-57361).

**To: Academic Editor, Editor-in-Chief, PLOS ONE**

Dear Editor-in-Chief or *Academic Editor*

We sincerely thank you for the opportunity to revise our manuscript. We also greatly appreciate the insightful comments and constructive suggestions provided by the reviewers. We have carefully addressed all the points raised, as detailed below:

All the suggestions found important and action is made

Responses for the Academic Editor

Journal Requirements:

In your revision ensure you cite all your sources (including your own works), and quote or rephrase any duplicated text outside the methods section.

Answer: We did as require and suggested

3. In the ethics statement in the Methods, you have specified that verbal consent was obtained. Please provide additional details regarding how this consent was documented and witnessed, and state whether this was approved by the IRB

Answer: We did as require and suggested

4. Your ethics statement should only appear in the Methods section of your manuscript. If your ethics statement is written in any section besides the Methods, please move it to the Methods section and delete it from any other section. Please ensure that your ethics statement is included in your manuscript, as the ethics statement entered the online submission form will not be published alongside your manuscript.

Answer: We did as require and suggested

5. If the reviewer comments include a recommendation to cite specific previously published works, please review and evaluate these publications to determine whether they are relevant and should be cited. There is no requirement to cite these works unless the editor has indicated otherwise.

Answer: We did as require and suggested

**Additional Editor Comments:**

The manuscript requires major revision. Although one reviewer recommended rejection, the main concerns identified are correctable and relate to methodology, statistical analysis, data clarity, ethics, structure, language, and references. The authors are invited to substantially revise the manuscript, noting that inadequate revision may lead to rejection.

Answer: We did a massive effort to improve the manuscript as require and suggested

**Reviewer #1**:

Rev’s opinion about the MS (PONE-D-25-54361), entitled „Scaling Field Pea Technology in Dryland Areas through a Cluster Approach: The Case of Waghimra Zone, Ethiopia”

An interesting agronomic challenge is the introduction of a new technology that contradicts established field practices in a given area.

Detailed remarks and recommendations:

**Abstract:**

The four-year study makes the vintage effect, which makes field studies difficult and unpredictable, suitable for drawing scientific conclusions. However, I miss the brief 1-2 sentence abstract presentation of the technology underlying the experiments.

Answer: We have added important introductory sentences as require and suggested

**Introduction:**

L39, the taxonomic affiliation of the pea must be given (Fabaceae OR Papilionaceous)

Answer: We have added as suggested

The cultivation location is limited by the climatic and soil requirements of the pea. I believe that the abiotic requirements of the pea, the characteristics of the cultivation intensity, and the main agrotechnical elements should be presented in 2-3 sentences. In the context of this, it is possible to judge the adaptability of the proposed technology.

Answer: We added important sentences as require and suggested

The world's leading protein crop is soybean. Wouldn't it be a potential competitor to the pea? Why pea? These questions should be answered in accordance with the criteria for preparing a scientific literature review. We cited references

Answer: Of course you are right but in the study area soybean is unknown and even no any adaptation trial is made.

In L53, the sentence "Therefore, there was......." should be deleted or its content should be changed, because there is no place for inferred thoughts here.

Answer: We have accepted and changed as suggested.

In addition, the entire chapter is MM-type, so it needs to be moved or deleted

The goals to be stated at the end of this chapter need to be formulated more specifically, clearly and separately.

Answer: We accepted and changed as suggested

**Material and Methods**

How were the farmers selected? We checked

Incoherent sentence: "Was the farming practice carried out in accordance with the regulations? What does this mean?

L80.81 to be deleted. I deleted or checked

Answer: accepted and we have deleted Line 80 and L81

Absolutely insufficient description of the examination.

Answer: we improved to be sufficient in the revised manuscript

A separate chapter should discuss the new technology tested, present its details! A separate subchapter would also be needed to present the selected farms! (What criteria system was used to select them? A separate chapter would be needed to present the climate conditions of each year!

Answer: we improved as per the suggestion in the revised manuscript

The description of the data collection is incomplete, there is no statistical evaluation!

Answer: we improved and made it complete in the revised manuscript

There is no place for a specially formatted citation or footnote in the manuscript.

The number of reviewed literatures is insufficient. In the absence of these basic antecedents, the results cannot be objectively evaluated further.

Answer: we have improved the text and added relevant references. Additionally, the footnote has been removed, and the information previously contained in the footnote has been integrated into the appropriate sections of the manuscript for better clarity and readability.

**Reviewer #2**

This manuscript presents a multi-year (2019–2022) scaling-up study of an improved field pea variety (“Yewagnesh”) using a cluster-based extension approach in dry-land areas of the Wag-Himra zone, Ethiopia. The topic is relevant and appropriate for PLOS ONE, particularly given the journal’s interest in applied agricultural research, technology dissemination, and development-oriented studies. The study demonstrates clear yield advantages, strong farmer interest, and meaningful stakeholder engagement. The manuscript is generally well structured, and the results are coherent.

However, several methodological clarifications, data transparency issues, and language improvements are required before the manuscript can be considered for publication.

1. Study design and comparison framework need clearer justification.

The manuscript reports a 71.1% yield advantage of the improved technology over local practices. However, it is not fully clear how yield comparisons were standardized across years and locations.

Answer: we improved and all raised issues are adressed in the revised manuscript

2. Statistical analysis is largely descriptive and should be strengthened

The manuscript relies primarily on descriptive statistics (means, percentages, Likert scores). While this is acceptable for extension research, the authors should justify the absence of inferential statistics or, where possible, include them.

Answer: we improved and all raised issues are addressed as per the suggetion in the revised manuscript

3**.** Extension gap calculation requires clearer linkage to data sources

The extension gap (EG) calculation uses yield data partly drawn from previously published studies. This raises concerns about comparability.

Answer: we improved and all raised issues are addressed as per the suggetion in the revised manuscript

5. Ethics statement needs expansion

The study involves human participants (farmers). While verbal consent is mentioned, the manuscript lacks:

Identification of an approving institutional body or ethics committee, or

A justification for why formal ethical approval was not required.

Answer: Justfication is provided. So, formal ethical approval was not required for this study because the research did not involve medical, clinical, or psychological experimentation on human subjects, nor did it include invasive procedures or the collection of sensitive personal data. Participation of farmers was voluntary and based on their willingness to implement and evaluate the improved field pea technologies on their own farms. The study therefore posed no physical, social, or economic risk to participants and followed standard ethical principles of voluntary participation and confidentiality, making formal institutional ethical approval unnecessary.

6. Language and clarity

The manuscript would benefit from professional English editing. Common issues include:

Repetition of phrases, grammatical inconsistencies, and informal or ambiguous expressions

Answer: we have made foremost improvement

7. Terminology consistency

Terms such as scaling-up, scaling-out, technology, and full package are sometimes used interchangeably. The authors should:

Define these terms clearly at first use

Apply them consistently throughout the manuscript

Answer: we made improvement as per the suggestion. We have maintained consistency in terminology, for example, using “scaling-up” uniformly across the manuscript.

8. Tables and formatting

Some tables lack clear captions or consistent formatting.

Percentages and sample sizes should be clearly linked in all tables.

Table 4 (SWOT analysis) would benefit from clearer alignment and explanation in the text.

Answer: we made improvement as per the suggestion. We have used clear captions and ensured consistent formatting across all tables.

9. Discussion could be better integrated with broader literature

While relevant studies are cited, the discussion would be strengthened by:

Comparing findings with similar scaling or cluster-based extension studies in dry-land systems.

Discussing transferability beyond the Wag-Himra zone.

Explicitly addressing limitations of the cluster approach.

Answer: we are challenged by lack of related literature, but we made improvement using the existing ones

**Reviewer #3:**

**Introduction**

Introduction is relevant and well written to introduce the subject clearly. However, the cited references are old. Not even one within the past 5 years. Does this mean that there is limited research around this subject to base your arguments on?

Answer: Of course there is limited research around this subject, but made improvement accordingly. We have revised and replaced the old citation with a new citation published within the past five years.

**Materials and Methods**

Line 71-72: The description of landholding is unclear and should specify whether the 32.5 ha refers to a collective cluster area or individual holdings. Does this mean that all 109 farmers share 34ha, please unpack it clearly?

Line 72: Clarify the criteria used for selecting the 109 farmers. And why only 22 female famers?

Answer: we made improvements to make it clear accordingly

Line 74: What is the meaning of ‘promoted’ in this context?

Answer: we made improvements to make it clear accordingly

Lines 87 to 89: The description of the questionnaire-based data collection lacks sufficient methodological detail. The rationale for selecting 30% of participants is not provided, and the implementation of systematic sampling (including list ordering and starting point) is unclear, just like in Line 72.

Answer: we made improvements to make it clear accordingly

**Results**

The SWOT analysis presented in Table 4 appears largely descriptive. The methodology used to generate and assign the SWOT components to the different actors is not clearly described, and several items reflect activities or personal perceptions rather than systemic strengths or weaknesses. This limits the analytical value of the SWOT in relation to the study objectives.”

Answer: we made improvements to make it clear

**Conclusion**

The recommendation to scale out the ‘Yewagnesh’ field pea variety is reasonable, but given the use of secondary data and descriptive analysis, it would benefit from a more cautious framing.

The study relies partly on secondary data from published literature to compute the extension gap. While this is acceptable in scaling-up assessments, the absence of a contemporaneous experimental control limits causal inference.

Answer: I made an important justification for the raised issues and worriers

**References:**

Generally, your references need to be reworked. Consistency is important in referencing, which lacks greatly in this case. Some examples below:

Line 227, check Russell. (1997). No initials, consistency.

Line 229 Tamene, Journal volume, number and page numbers missing.

Line 232 Yadav DB, no comma after surname and also journal name is abbreviated, different from most.

Line 216 Mihiretu, Journal name abbreviated.

Answer: We make the required justification

In **gerenral**:

- We incorporated the ethics statement only in the Methods section.
- All references have been cited in accordance with the journal guidelines
- Amhara Regional Agricultural Research Institute, Sekota Dryland Agricultural Research Center, Sekota, Ethiopia. This is our updated affilation.

We trust that these revisions meet the expectations of the reviewers and the editorial team. Thank you once again for your time and consideration.

**Sincerely,**
Melaku Asresu
