# Supplementary material for: Scaling field pea technology in dryland areas through a cluster approach: The case of Waghimra zone, Ethiopia
Source: PLoS One. 2026 Jul 7;21(7):e0353275. doi: 10.1371/journal.pone.0353275 (PMC13340809; doi:10.1371/journal.pone.0353275)
Supplement: Peer review history

# Peer review: Decision Letter 0

**DOI:** 10.1371/journal.pone.0353275.r001

**Authors:** Ayman A Swelum

29 Jan 2026

PONE-D-25-57361

Scaling Field Pea Technology in Dry-Land Areas through a Cluster Approach: The Case of Waghimra Zone, Ethiopia

PLOS One

Dear Dr. Asresu,

Thank you for submitting your manuscript to PLOS ONE. After careful consideration, we feel that it has merit but does not fully meet PLOS ONE’s publication criteria as it currently stands. Therefore, we invite you to submit a revised version of the manuscript that addresses the points raised during the review process.

Please submit your revised manuscript by Mar 15 2026 11:59PM. If you will need more time than this to complete your revisions, please reply to this message or contact the journal office at plosone@plos.org. When you're ready to submit your revision, log on to https://www.editorialmanager.com/pone/ and select the 'Submissions Needing Revision' folder to locate your manuscript file.

Please include the following items when submitting your revised manuscript:

- A letter that responds to each point raised by the academic editor and reviewer(s). You should upload this letter as a separate file labeled 'Response to Reviewers'.
- A marked-up copy of your manuscript that highlights changes made to the original version. You should upload this as a separate file labeled 'Revised Manuscript with Track Changes'.
- An unmarked version of your revised paper without tracked changes. You should upload this as a separate file labeled 'Manuscript'.

If you would like to make changes to your financial disclosure, please include your updated statement in your cover letter. Guidelines for resubmitting your figure files are available below the reviewer comments at the end of this letter.

If applicable, we recommend that you deposit your laboratory protocols in protocols.io to enhance the reproducibility of your results. Protocols.io assigns your protocol its own identifier (DOI) so that it can be cited independently in the future. For instructions see: https://journals.plos.org/plosone/s/submission-guidelines#loc-laboratory-protocols. Additionally, PLOS ONE offers an option for publishing peer-reviewed Lab Protocol articles, which describe protocols hosted on protocols.io. Read more information on sharing protocols at https://plos.org/protocols?utm_medium=editorial-email&utm_source=authorletters&utm_campaign=protocols.

We look forward to receiving your revised manuscript.

Kind regards,

Ayman A Swelum

Academic Editor

PLOS One

Journal Requirements:

When submitting your revision, we need you to address these additional requirements.

1.Please ensure that your manuscript meets PLOS ONE's style requirements, including those for file naming. The PLOS ONE style templates can be found at

https://journals.plos.org/plosone/s/file?id=wjVg/PLOSOne_formatting_sample_main_body.pdf and

https://journals.plos.org/plosone/s/file?id=ba62/PLOSOne_formatting_sample_title_authors_affiliations.pdf

2. We noticed you have some minor occurrence of overlapping text with the following previous publication(s), which needs to be addressed:

https://www.researchgate.net/publication/369691002_PEARL_MILLET_THE_HOPE_OF_FOOD_SECURITY_IN_MARGINAL_ARID_TROPICS_IMPLICATIONS_FOR_DIVERSIFYING_LIMITED_CROPPING_SYSTEMS

http://www.agrifoodscience.com/index.php/TURJAF/article/download/2857/1300

In your revision ensure you cite all your sources (including your own works), and quote or rephrase any duplicated text outside the methods section. Further consideration is dependent on these concerns being addressed.

3. In the ethics statement in the Methods, you have specified that verbal consent was obtained. Please provide additional details regarding how this consent was documented and witnessed, and state whether this was approved by the IRB

4. Your ethics statement should only appear in the Methods section of your manuscript. If your ethics statement is written in any section besides the Methods, please move it to the Methods section and delete it from any other section. Please ensure that your ethics statement is included in your manuscript, as the ethics statement entered into the online submission form will not be published alongside your manuscript.

5. If the reviewer comments include a recommendation to cite specific previously published works, please review and evaluate these publications to determine whether they are relevant and should be cited. There is no requirement to cite these works unless the editor has indicated otherwise.

Additional Editor Comments:

**T** he manuscript requires **major revision** . Although one reviewer recommended rejection, the main concerns identified are correctable and relate to methodology, statistical analysis, data clarity, ethics, structure, language, and references. The authors are invited to substantially revise the manuscript, noting that inadequate revision may lead to rejection.

[Note: HTML markup is below. Please do not edit.]

Reviewers' comments:

Reviewer's Responses to Questions

**Comments to the Author**

1. Is the manuscript technically sound, and do the data support the conclusions?

The manuscript must describe a technically sound piece of scientific research with data that supports the conclusions. Experiments must have been conducted rigorously, with appropriate controls, replication, and sample sizes. The conclusions must be drawn appropriately based on the data presented.

Reviewer #1: No

Reviewer #2: Partly

Reviewer #3: Yes

**********

2. Has the statistical analysis been performed appropriately and rigorously?

Reviewer #1: No

Reviewer #2: Yes

Reviewer #3: Yes

**********

3. Have the authors made all data underlying the findings in their manuscript fully available?

The PLOS Data policy requires authors to make all data underlying the findings described in their manuscript fully available without restriction, with rare exception (please refer to the Data Availability Statement in the manuscript PDF file). The data should be provided as part of the manuscript or its supporting information, or deposited to a public repository. For example, in addition to summary statistics, the data points behind means, medians and variance measures should be available. If there are restrictions on publicly sharing data—e.g. participant privacy or use of data from a third party—those must be specified.

Reviewer #1: No

Reviewer #2: No

Reviewer #3: No

**********

4. Is the manuscript presented in an intelligible fashion and written in standard English?

PLOS ONE does not copyedit accepted manuscripts, so the language in submitted articles must be clear, correct, and unambiguous. Any typographical or grammatical errors should be corrected at revision, so please note any specific errors here.

Reviewer #1: Yes

Reviewer #2: No

Reviewer #3: Yes

**********

5. Review Comments to the Author

Please use the space provided to explain your answers to the questions above. You may also include additional comments for the author, including concerns about dual publication, research ethics, or publication ethics. (Please upload your review as an attachment if it exceeds 20,000 characters)

Reviewer #1: Rev’s opinion about the MS (PONE-D-25-54361), entitled “Scaling Field Pea Technology in Dry-Land Areas through a Cluster Approach: The Case of Waghimra Zone, Ethiopia”

An interesting agronomic challenge is the introduction of a new technology that contradicts established field practices in a given area.

Detailed remarks and recommendations:

Abstract:

The four-year study makes the vintage effect, which makes field studies difficult and unpredictable, suitable for drawing scientific conclusions. However, I miss the brief 1-2 sentence abstract presentation of the technology underlying the experiments.

Introduction:

L39, the taxonomic affiliation of the pea must be given (Fabaceae OR Papilionaceae)

The cultivation location is limited by the climatic and soil requirements of the pea. I believe that the abiotic requirements of the pea, the characteristics of the cultivation intensity, and the main agrotechnical elements should be presented in 2-3 sentences. In the context of this, it is possible to judge the adaptability of the proposed technology

The world's leading protein crop is soybean. Wouldn't it be a potential competitor to the pea? Why pea? These questions should be answered in accordance with the criteria for preparing a scientific literature review.

In L53, the sentence "Therefore, there was......." should be deleted or its content should be changed, because there is no place for inferred thoughts here.

In addition, the entire chapter is MM-type, so it needs to be moved or deleted

The goals to be stated at the end of this chapter need to be formulated more specifically, clearly and separately.

Material and Methods

How were the farmers selected?

Incoherent sentence: "Was the farming practice carried out in accordance with the regulations? What does this mean?

L80.81 to be deleted

Absolutely insufficient description of the examination

A separate chapter should discuss the new technology tested, present its details! A separate subchapter would also be needed to present the selected farms! (What criteria system was used to select them? A separate chapter would be needed to present the climate conditions of each year! ű

The description of the data collection is incomplete, there is no statistical evaluation!

There is no place for a specially formatted citation or footnote in the manuscript.

The number of reviewed literature is insufficient..

In the absence of these basic antecedents, the results cannot be objectively evaluated further. The examination does not meet the basic expectations of scientific papers.

Due to all these fundamental errors, I do not consider the manuscript correctable. For this reason, I decide to reject it

Reviewer #2: This manuscript presents a multi-year (2019–2022) scaling-up study of an improved field pea variety (“Yewagnesh”) using a cluster-based extension approach in dry-land areas of the Wag-Himra zone, Ethiopia. The topic is relevant and appropriate for PLOS ONE, particularly given the journal’s interest in applied agricultural research, technology dissemination, and development-oriented studies. The study demonstrates clear yield advantages, strong farmer interest, and meaningful stakeholder engagement. The manuscript is generally well structured, and the results are coherent. However, several methodological clarifications, data transparency issues, and language improvements are required before the manuscript can be considered for publication.

1. Study design and comparison framework need clearer justification

The manuscript reports a 71.1% yield advantage of the improved technology over local practices. However, it is not fully clear how yield comparisons were standardized across years and locations.

Specifically, the authors should clarify:

Whether yield data were averaged across years or analyzed year-by-year.

How environmental variability (rainfall differences across seasons) was accounted for.

Whether the “local practice” plots were managed by the same farmers under comparable conditions.

2. Statistical analysis is largely descriptive and should be strengthened

The manuscript relies primarily on descriptive statistics (means, percentages, Likert scores). While this is acceptable for extension research, the authors should justify the absence of inferential statistics or, where possible, include them.

Suggestions:

Include basic statistical tests (e.g., t-tests or non-parametric alternatives) to support yield comparisons.

Clearly report variance components when comparing improved vs. local practices.

Explicitly state assumptions and limitations if inferential analysis is not feasible.

3. Extension gap calculation requires clearer linkage to data sources

The extension gap (EG) calculation uses yield data partly drawn from previously published studies. This raises concerns about comparability.

The authors should:

Clearly separate data derived from the current scaling-up activity and secondary data taken from earlier demonstrations.

Explain why combining these datasets is methodologically valid.

Discuss potential biases introduced by using historical demonstration data for EG estimation.

5. Ethics statement needs expansion

The study involves human participants (farmers). While verbal consent is mentioned, the manuscript lacks:

Identification of an approving institutional body or ethics committee, or

A justification for why formal ethical approval was not required.

6. Language and clarity

The manuscript would benefit from professional English editing. Common issues include:

Repetition of phrases

Grammatical inconsistencies

Informal or ambiguous expressions

7. Terminology consistency

Terms such as scaling-up, scaling-out, technology, and full package are sometimes used interchangeably. The authors should:

Define these terms clearly at first use

Apply them consistently throughout the manuscript

8. Tables and formatting

Some tables lack clear captions or consistent formatting.

Percentages and sample sizes should be clearly linked in all tables.

Table 4 (SWOT analysis) would benefit from clearer alignment and explanation in the text.

9. Discussion could be better integrated with broader literature

While relevant studies are cited, the discussion would be strengthened by:

Comparing findings with similar scaling or cluster-based extension studies in dry-land systems.

Discussing transferability beyond the Wag-Himra zone.

Explicitly addressing limitations of the cluster approach.

Reviewer #3: Introduction

Introduction is relevant and well written to introduce the subject clearly. However, the cited references are old. Not even one within the past 5 years. Does this mean that there is limited research around this subject to base your arguments on?

Materials and Methods

Line 71-72: The description of landholding is unclear and should specify whether the 32.5 ha refers to a collective cluster area or individual holdings. Does this mean that all 109 farmers share 34ha, please unpack it clearly?

Line 72: Clarify the criteria used for selecting the 109 farmers. And why only 22 female famers?

Line 74: What is the meaning of ‘promoted’ in this context?

Lines 87 to 89: The description of the questionnaire-based data collection lacks sufficient methodological detail. The rationale for selecting 30% of participants is not provided, and the implementation of systematic sampling (including list ordering and starting point) is unclear, just like in Line 72.

Results

The SWOT analysis presented in Table 4 appears largely descriptive. The methodology used to generate and assign the SWOT components to the different actors is not clearly described, and several items reflect activities or personal perceptions rather than systemic strengths or weaknesses. This limits the analytical value of the SWOT in relation to the study objectives.”

Conclusion

The recommendation to scale out the ‘Yewagnesh’ field pea variety is reasonable, but given the use of secondary data and descriptive analysis, it would benefit from a more cautious framing.

The study relies partly on secondary data from published literature to compute the extension gap. While this is acceptable in scaling-up assessments, the absence of a contemporaneous experimental control limits causal inference.

References:

Generally, your references need to be reworked. Consistecy is important in referencing, which lacks greatly in this case. Some examples below:

Line 227, check Russell. (1997). No initials, consistency.

Line 229 Tamene, Journal volume, number and page numbers missing.

Line 232 Yadav DB, no comma after surname and also journal name is abbreviated, different from most.

Line 216 Mihiretu, Journal name abbreviated.

I suggest a rework on the reference lis vs cited references in text.

**********

6. PLOS authors have the option to publish the peer review history of their article (what does this mean?). If published, this will include your full peer review and any attached files.

If you choose “no”, your identity will remain anonymous but your review may still be made public.

**Do you want your identity to be public for this peer review?**  For information about this choice, including consent withdrawal, please see our Privacy Policy.

Reviewer #1: No

Reviewer #2: No

Reviewer #3: No

**********

[NOTE: If reviewer comments were submitted as an attachment file, they will be attached to this email and accessible via the submission site. Please log into your account, locate the manuscript record, and check for the action link "View Attachments". If this link does not appear, there are no attachment files.]

To ensure your figures meet our technical requirements, please review our figure guidelines: https://journals.plos.org/plosone/s/figures

You may also use PLOS’s free figure tool, NAAS, to help you prepare publication quality figures: https://journals.plos.org/plosone/s/figures#loc-tools-for-figure-preparation.

NAAS will assess whether your figures meet our technical requirements by comparing each figure against our figure specifications.

Attachment Submitted filename: PONE-D-25-57361_reviewer-1_AB.pdf

# Author comment: Author response to Decision Letter 1

**DOI:** 10.1371/journal.pone.0353275.r002

15 Mar 2026

Date: 10/03/2026

Author Response to Reviewers

Manuscript Title: Scaling Field Pea Technology in Dry-Land Areas through a Cluster Approach: The Case of Waghimra Zone, Ethiopia (PONE-D-25-57361).

To: Editor-in-Chief, PLOS ONE

Dear Editor-in-Chief,

We sincerely thank you for the opportunity to revise our manuscript. We also greatly appreciate the insightful comments and constructive suggestions provided by the reviewers. We have carefully addressed all the points raised, as detailed below:

Reviewer #1

1. We have revised and edited the taxonomic affiliation of pea to Fabaceae in the introduction section

2. We have addressed the question, in accordance with the scientific literature, of why pea is considered a protein-source crop and a potential competitor to other protein crops.

3. We have paraphrased and revised the content in Line 53 according to the reviewer’s comments.

4. We have formulated the goals specifically, clearly, and separately, to be stated at the end of the Introduction section.

5. We have clearly stated how the farmers were selected in the Materials and Methods section.

6. We have deleted L80 and L81 according to the reviewers’ comment.

7. We have revised and paraphrased the data collection sections in accordance with the reviewers’ comments.

8. We have removed the footnote and incorporated its content into the appropriate sections of the manuscript.

9. We have included additional citations and references to reflect the reviewed literature.

Reviewer #2

1. The study reports a 71.1% yield advantage of the improved technology over local practices, based on yield data averaged across years.

2. The ‘local practice’ plots were adjacent to the improved practice plots and were managed by the same farmers under comparable conditions.

3. Since the study focuses on extension research, descriptive statistics (means, percentages, Likert scores) were considered sufficient, and inferential statistics were not applied.

4. Although the extension gap (EG) calculation partly relies on previously published yield data, we consider it valid because similar farming systems were used during the demonstration phase, and the scaling was carried out on comparable farms.

5. We believe that combining Extension Gap datasets is methodologically valid. Incorporating the Extension Gap in the methodology allows us to demonstrate both the effectiveness of improved varieties compared to local varieties and the yield loss resulting from improper or incomplete application of extension recommendations.

6. We have revised the text to remove repetitive phrases and clarify informal or ambiguous expressions.

7. We have maintained consistency in terminology, for example, using “scaling-up” uniformly across the manuscript.

8. We have used clear captions and ensured consistent formatting across all tables.

9. We have compared our findings with those from similar scaling- or cluster-based extension studies in dryland systems.

10. Formal ethical approval was not required for this study because the research did not involve medical, clinical, or psychological experimentation on human subjects, nor did it include invasive procedures or the collection of sensitive personal data. Participation of farmers was voluntary and based on their willingness to implement and evaluate the improved field pea technologies on their own farms. The study therefore posed no physical, social, or economic risk to participants and followed standard ethical principles of voluntary participation and confidentiality, making formal institutional ethical approval unnecessary.

Reviewer #3

1. We have revised and replaced the old citation with a new citation published within the past five years.

2. Lines 71–72 clarify that the total land area of 32.5 hectares is collectively shared among the 109 participating farmers.

3. Line 72: Farmers were purposively selected, in collaboration with the District Office, to participate in the scaling-up initiative, implement the technology following recommended agronomic practices, and serve as model farmers for subsequent large-scale dissemination efforts. The proportion of female participants corresponds to the prevailing gender distribution of landholding and field pea production in the study area.

4. Line 74: The term “promoted” refers to the dissemination or introduction of the technology.

In gerenral:

All references have been cited in accordance with the journal guidelines

Amhara Regional Agricultural Research Institute, Sekota Dryland Agricultural Research Center, Sekota, Ethiopia. This is our updated affilation.

We trust that these revisions meet the expectations of the reviewers and the editorial team. Thank you once again for your time and consideration.

Sincerely,

Melaku Asresu

Attachment Submitted filename: Response to Reviewers 2026.docx

# Peer review: Decision Letter 1

**DOI:** 10.1371/journal.pone.0353275.r003

**Authors:** Ayman A Swelum

30 Apr 2026

PONE-D-25-57361R1

Scaling Field Pea Technology in Dryland Areas through a Cluster Approach: The Case of Waghimra Zone, Ethiopia

PLOS One

Dear Dr. Asresu,

Thank you for submitting your manuscript to PLOS ONE. After careful consideration, we feel that it has merit but does not fully meet PLOS ONE’s publication criteria as it currently stands. Therefore, we invite you to submit a revised version of the manuscript that addresses the points raised during the review process.

==============================

**ACADEMIC EDITOR: Please respond carefully for all reviewers comments**

==============================

Please submit your revised manuscript by Jun 14 2026 11:59PM. If you will need more time than this to complete your revisions, please reply to this message or contact the journal office at plosone@plos.org. When you're ready to submit your revision, log on to https://www.editorialmanager.com/pone/ and select the 'Submissions Needing Revision' folder to locate your manuscript file.

Please include the following items when submitting your revised manuscript:

- A letter that responds to each point raised by the academic editor and reviewer(s). You should upload this letter as a separate file labeled 'Response to Reviewers'.
- A marked-up copy of your manuscript that highlights changes made to the original version. You should upload this as a separate file labeled 'Revised Manuscript with Track Changes'.
- An unmarked version of your revised paper without tracked changes. You should upload this as a separate file labeled 'Manuscript'.

If you would like to make changes to your financial disclosure, please include your updated statement in your cover letter. Guidelines for resubmitting your figure files are available below the reviewer comments at the end of this letter.

If applicable, we recommend that you deposit your laboratory protocols in protocols.io to enhance the reproducibility of your results. Protocols.io assigns your protocol its own identifier (DOI) so that it can be cited independently in the future. For instructions see: https://journals.plos.org/plosone/s/submission-guidelines#loc-laboratory-protocols. Additionally, PLOS ONE offers an option for publishing peer-reviewed Lab Protocol articles, which describe protocols hosted on protocols.io. Read more information on sharing protocols at https://plos.org/protocols?utm_medium=editorial-email&utm_source=authorletters&utm_campaign=protocols.

As the corresponding author, your ORCID iD is verified in the submission system and will appear in the published article. PLOS supports the use of ORCID, and we encourage all coauthors to register for an ORCID iD and use it as well. Please encourage your coauthors to verify their ORCID iD within the submission system before final acceptance, as unverified ORCID iDs will not appear in the published article. *Only*  the individual author can complete the verification step; PLOS staff *cannot*  verify ORCID iDs on behalf of authors.

We look forward to receiving your revised manuscript.

Kind regards,

Ayman A Swelum

Academic Editor

PLOS One

Journal Requirements:

If the reviewer comments include a recommendation to cite specific previously published works, please review and evaluate these publications to determine whether they are relevant and should be cited. There is no requirement to cite these works unless the editor has indicated otherwise.

[Note: HTML markup is below. Please do not edit.]

Reviewers' comments:

Reviewer's Responses to Questions

**Comments to the Author**

1. If the authors have adequately addressed your comments raised in a previous round of review and you feel that this manuscript is now acceptable for publication, you may indicate that here to bypass the “Comments to the Author” section, enter your conflict of interest statement in the “Confidential to Editor” section, and submit your "Accept" recommendation.

Reviewer #1: (No Response)

Reviewer #3: (No Response)

**********

2. Is the manuscript technically sound, and do the data support the conclusions?

The manuscript must describe a technically sound piece of scientific research with data that supports the conclusions. Experiments must have been conducted rigorously, with appropriate controls, replication, and sample sizes. The conclusions must be drawn appropriately based on the data presented.

Reviewer #1: Partly

Reviewer #3: Yes

**********

3. Has the statistical analysis been performed appropriately and rigorously?

Reviewer #1: Yes

Reviewer #3: Yes

**********

4. Have the authors made all data underlying the findings in their manuscript fully available?

The PLOS Data policy requires authors to make all data underlying the findings described in their manuscript fully available without restriction, with rare exception (please refer to the Data Availability Statement in the manuscript PDF file). The data should be provided as part of the manuscript or its supporting information, or deposited to a public repository. For example, in addition to summary statistics, the data points behind means, medians and variance measures should be available. If there are restrictions on publicly sharing data—e.g. participant privacy or use of data from a third party—those must be specified.

Reviewer #1: Yes

Reviewer #3: Yes

**********

5. Is the manuscript presented in an intelligible fashion and written in standard English?

PLOS ONE does not copyedit accepted manuscripts, so the language in submitted articles must be clear, correct, and unambiguous. Any typographical or grammatical errors should be corrected at revision, so please note any specific errors here.

Reviewer #1: Yes

Reviewer #3: Yes

**********

6. Review Comments to the Author

Please use the space provided to explain your answers to the questions above. You may also include additional comments for the author, including concerns about dual publication, research ethics, or publication ethics. (Please upload your review as an attachment if it exceeds 20,000 characters)

Reviewer #1: The manuscript has undergone a thorough revision compared to the first, original manuscript. I received satisfactory answers to my formulated questions. Thus, the value of the article has increased significantly. Although I still maintain that the scientific value and uniqueness of the manuscript are less prominent and its literary support and supplementation are needed, and further confrontations with existing international studies would also increase the value of the work.

Reviewer #3: My concerns are still on the description of farmer and farm selection, which remains unclear and methodologically inconsistent. The manuscript alternates between purposive selection, “determination by chance,” and criteria such as accessibility, farmer interest, and budget constraints, without clearly defining the sampling framework. This creates ambiguity regarding whether the study follows a qualitative/participatory approach or a quantitative experimental design. I am really struggling to place it from reading sections under lines 107 and 121.

As currently presented, the methodological basis for farmer selection and study design is insufficiently justified. While the work has merit and could contribute meaningfully to the literature on technology scaling under real farming conditions, significant revision is required to address these methodological and presentation concerns.

**********

7. PLOS authors have the option to publish the peer review history of their article (what does this mean?). If published, this will include your full peer review and any attached files.

If you choose “no”, your identity will remain anonymous but your review may still be made public.

**Do you want your identity to be public for this peer review?**  For information about this choice, including consent withdrawal, please see our Privacy Policy.

Reviewer #1: No

Reviewer #3: No

**********

[NOTE: If reviewer comments were submitted as an attachment file, they will be attached to this email and accessible via the submission site. Please log into your account, locate the manuscript record, and check for the action link "View Attachments". If this link does not appear, there are no attachment files.]

To ensure your figures meet our technical requirements, please review our figure guidelines: https://journals.plos.org/plosone/s/figures

You may also use PLOS’s free figure tool, NAAS, to help you prepare publication quality figures: https://journals.plos.org/plosone/s/figures#loc-tools-for-figure-preparation.

NAAS will assess whether your figures meet our technical requirements by comparing each figure against our figure specifications.

# Author comment: Author response to Decision Letter 2

**DOI:** 10.1371/journal.pone.0353275.r004

20 May 2026

We are pleased to submit the revised version of our manuscript entitled “Scaling Field Pea Technology in Dryland Areas through a Cluster Approach: The Case of Waghimra Zone, Ethiopia.” We have carefully addressed the reviewers’ comments by strengthening the comparative discussion with international studies, clarifying methodological details, and refining the presentation of results, limitations, and conclusions. The manuscript has been thoroughly revised to improve clarity, coherence, and alignment with journal standards.

We believe the revisions have significantly enhanced the quality of the paper and hope it will now meet the requirements for publication.

Attachment Submitted filename: Response to Reviewers.docx

# Peer review: Decision Letter 2

**DOI:** 10.1371/journal.pone.0353275.r005

**Authors:** Ayman A Swelum

22 Jun 2026

Scaling Field Pea Technology in Dryland Areas through a Cluster Approach: The Case of Waghimra Zone, Ethiopia

PONE-D-25-57361R2

Dear Dr. Asresu,

We’re pleased to inform you that your manuscript has been judged scientifically suitable for publication and will be formally accepted for publication once it meets all outstanding technical requirements.

Within one week, you’ll receive an e-mail detailing the required amendments. When these have been addressed, you’ll receive a formal acceptance letter and your manuscript will be scheduled for publication.

An invoice will be generated when your article is formally accepted. Please note, if your institution has a publishing partnership with PLOS and your article meets the relevant criteria, all or part of your publication costs will be covered. Please make sure your user information is up-to-date by logging into Editorial Manager at Editorial Manager® and clicking the ‘Update My Information' link at the top of the page. For questions related to billing, please contact billing support.

If your institution or institutions have a press office, please notify them about your upcoming paper to help maximize its impact. If they’ll be preparing press materials, please inform our press team as soon as possible -- no later than 48 hours after receiving the formal acceptance. Your manuscript will remain under strict press embargo until 2 pm Eastern Time on the date of publication. For more information, please contact onepress@plos.org.

Kind regards,

Ayman A Swelum

Academic Editor

PLOS One

Additional Editor Comments (optional):

Reviewers' comments:

Reviewer's Responses to Questions

**Comments to the Author**

1. If the authors have adequately addressed your comments raised in a previous round of review and you feel that this manuscript is now acceptable for publication, you may indicate that here to bypass the “Comments to the Author” section, enter your conflict of interest statement in the “Confidential to Editor” section, and submit your "Accept" recommendation.

Reviewer #1: (No Response)

Reviewer #3: All comments have been addressed

**********

2. Is the manuscript technically sound, and do the data support the conclusions?

The manuscript must describe a technically sound piece of scientific research with data that supports the conclusions. Experiments must have been conducted rigorously, with appropriate controls, replication, and sample sizes. The conclusions must be drawn appropriately based on the data presented.

Reviewer #1: Yes

Reviewer #3: Yes

**********

3. Has the statistical analysis been performed appropriately and rigorously?

Reviewer #1: Yes

Reviewer #3: Yes

**********

4. Have the authors made all data underlying the findings in their manuscript fully available?

The PLOS Data policy requires authors to make all data underlying the findings described in their manuscript fully available without restriction, with rare exception (please refer to the Data Availability Statement in the manuscript PDF file). The data should be provided as part of the manuscript or its supporting information, or deposited to a public repository. For example, in addition to summary statistics, the data points behind means, medians and variance measures should be available. If there are restrictions on publicly sharing data—e.g. participant privacy or use of data from a third party—those must be specified.

Reviewer #1: Yes

Reviewer #3: Yes

**********

5. Is the manuscript presented in an intelligible fashion and written in standard English?

PLOS ONE does not copyedit accepted manuscripts, so the language in submitted articles must be clear, correct, and unambiguous. Any typographical or grammatical errors should be corrected at revision, so please note any specific errors here.

Reviewer #1: Yes

Reviewer #3: Yes

**********

6. Review Comments to the Author

Please use the space provided to explain your answers to the questions above. You may also include additional comments for the author, including concerns about dual publication, research ethics, or publication ethics. (Please upload your review as an attachment if it exceeds 20,000 characters)

Reviewer #1: Dear Authors, thanks a lo for your work and responses . The authors have made the necessary corrections, based on which the manuscript is acceptable.

Reviewer #3: The concerns I raised under methodology on the description of farmer and farm selection were addressed. The answers are satisfactory, and the manuscript can be accepted.

**********

7. PLOS authors have the option to publish the peer review history of their article (what does this mean?). If published, this will include your full peer review and any attached files.

If you choose “no”, your identity will remain anonymous but your review may still be made public.

**Do you want your identity to be public for this peer review?**  For information about this choice, including consent withdrawal, please see our Privacy Policy.

Reviewer #1: **Yes:**  Sándor Keszthelyi

Reviewer #3: No

**********

# Editor report: Acceptance letter

**DOI:** 10.1371/journal.pone.0353275.r006

**Authors:** Ayman A Swelum

PONE-D-25-57361R2

PLOS One

Dear Dr. Asresu,

I'm pleased to inform you that your manuscript has been deemed suitable for publication in PLOS One. Congratulations! Your manuscript is now being handed over to our production team.

At this stage, our production department will prepare your paper for publication. This includes ensuring the following:

* All references, tables, and figures are properly cited

* All relevant supporting information is included in the manuscript submission,

* There are no issues that prevent the paper from being properly typeset

You will receive further instructions from the production team, including instructions on how to review your proof when it is ready. Please keep in mind that we are working through a large volume of accepted articles, so please give us a few days to review your paper and let you know the next and final steps.

Lastly, if your institution or institutions have a press office, please let them know about your upcoming paper now to help maximize its impact. If they'll be preparing press materials, please inform our press team within the next 48 hours. Your manuscript will remain under strict press embargo until 2 pm Eastern Time on the date of publication. For more information, please contact onepress@plos.org.

You will receive an invoice from PLOS for your publication fee after your manuscript has reached the completed accept phase. If you receive an email requesting payment before acceptance or for any other service, this may be a phishing scheme. Learn how to identify phishing emails and protect your accounts at https://explore.plos.org/phishing.

If we can help with anything else, please email us at customercare@plos.org.

Thank you for submitting your work to PLOS ONE and supporting open access.

Kind regards,

PLOS ONE Editorial Office Staff

on behalf of

Professor Ayman A Swelum

Academic Editor

PLOS One
